# Distinctive chemotactic responses of three marine herbivore protists to DMSP and related compounds

**DOI:** 10.1093/ismejo/wrae130

**Published:** 2024-07-12

**Authors:** Queralt Güell-Bujons, Medea Zanoli, Idan Tuval, Albert Calbet, Rafel Simó

**Affiliations:** Institut de Ciències del Mar, ICM-CSIC, 08003 Barcelona, Catalonia, Spain; Departament de Genètica i Microbiologia, Universitat Autònoma de Barcelona, 08193 Cerdanyola del Vallès, Catalonia, Spain; Institut Mediterrani d’Estudis Avançats, IMEDEA (UIB-CSIC), 07190 Esporles, Mallorca, Spain; Institut Mediterrani d’Estudis Avançats, IMEDEA (UIB-CSIC), 07190 Esporles, Mallorca, Spain; Institut de Ciències del Mar, ICM-CSIC, 08003 Barcelona, Catalonia, Spain; Institut de Ciències del Mar, ICM-CSIC, 08003 Barcelona, Catalonia, Spain

**Keywords:** chemotaxis, Oxyrrhis, Gyrodinium, Karlodinium, DMSP, DMS, acrylate, grazing, microzooplankton, capillary assays

## Abstract

Marine planktonic predator–prey interactions occur in microscale seascapes, where diffusing chemicals may act either as chemotactic cues that enhance or arrest predation, or as elemental resources that are complementary to prey ingestion. The phytoplankton osmolyte dimethylsulfoniopropionate (DMSP) and its degradation products dimethylsulfide (DMS) and acrylate are pervasive compounds with high chemotactic potential, but there is a longstanding controversy over whether they act as grazing enhancers or deterrents. Here, we investigated the chemotactic responses of three herbivorous dinoflagellates to point-sourced, microscale gradients of dissolved DMSP, DMS, and acrylate. We found no evidence for acrylate being a chemotactic repellent and observed a weak attractor role of DMS. DMSP behaved as a strong chemoattractor whose potential for grazing facilitation through effects on swimming patterns and aggregation depends on the grazer’s feeding mode and ability to incorporate DMSP. Our study reveals that predation models will fail to predict grazing impacts unless they incorporate chemotaxis-driven searching and finding of prey.

## Introduction

Emerging marine ecosystem properties, like nutrient recycling and carbon and energy fluxes, can be quantified at the meso- and regional scales using biogeochemical tracers and proxies. However, true comprehension lies in delving into the microscale realm, where planktonic microorganisms thrive and interact [1–3]. In this dynamic environment, marine microbial interactions are often driven by chemical cues, which play crucial roles in feeding behaviour [2, 4], cooperation and competition strategies [5, 6], and grazing relationships [7, 8]. Within this playground of planktonic chemical cross-talking, observed chemotactic responses of motile microorganisms have multiple potential ecological implications still to be discovered. Microzooplankton account for most of the daily grazing on phytoplankton across the oceans [5]. Grazing constitutes a multifaceted predator–prey interaction in which chemical cues, alongside other factors such as nutritional quality, behaviour, and defensive responses of the prey, play crucial roles [6–8]. Nevertheless, the extent to which microzooplankton respond to microscale chemical gradients in terms of prey selection and preferential grazing on specific individuals or populations remains largely unresolved. If chemical-based prey selection is confirmed as a widespread behaviour, it would challenge the way grazing is modelled, which currently relies on neutral encounter rates defined by predator and prey densities [9], relative speeds, and the use of size-based frameworks [10].

Amongst the myriad compounds that can act as chemical cues for marine micrograzers [8, 11, 12], dimethylsulfoniopropionate (DMSP) and related compounds hold particular importance due to their pervasive presence in the pelagic ocean [13] and their chemotactic potential [14]. DMSP is produced as a cellular solute by most marine algae and many bacteria [15, 16], where it plays several physiological roles (osmolyte, antioxidant, metabolic overflow), particularly under stress conditions [13, 17]. Intracellular concentrations span orders of magnitude amongst taxa (undetectable to 100s mM), varying greatly with physiological responses to stressors [17–21], and reaching up to 10% of the total cell carbon in highly productive microalgae such as haptophytes and dinoflagellates [16, 17]. Taxonomy and physiology-dependent variability results in a wide range of particulate DMSP concentrations in the ocean (5–4000 nM) [22, 23]. Overall, the global open ocean DMSP production is estimated to be 3.8 Pg C/year [24]. Within the marine environment, DMSP is partly converted into dimethylsulfide (DMS) and acrylate by DMSP-lyase enzymes hosted by phyto- and bacterioplankton species [25]. The conversion of DMSP to DMS initiates another cycle that expands beyond the ocean environment into the atmosphere. Global DMS emissions amount 15–40 Tg S year^−1^ and account for most of the biogenic atmospheric sulphate [26]. The release of DMSP, DMS, and acrylate occurs from various point sources, including phytoplankton cells experiencing oxidative stress [18] or excess of reduced sulphur [17], faecal pellets and other aggregates colonized by bacteria [27, 28], as well as algal cells disrupted by viral attack or grazing [29, 30]. The low background concentrations of dissolved DMSP typically measured in bulk seawater (1–25 nM; [22]) reflect its fast turnover (1–129 nM d^−1^, [22]). Actually, once released, these compounds may accumulate in the phycosphere or particle-sphere, which is the high viscosity zone surrounding cells or aggregates. This area is enriched with released organic molecules, with concentrations orders of magnitude higher than those found in the background seawater [2, 31]. Although modelled dissolved DMSP-related compound concentrations in the phycosphere may fluctuate from nanomolar to micromolar levels [32, 33], bursting cells, high DMSP-containing bursting cells or aggregates are more likely to generate patches with micromolar concentrations. Within and beyond this sphere, the diffusion of chemicals leads to the formation of submillimetre patches and gradients [1], providing a potential microscale landscape that influences trophic interactions [14, 26, 34].

Point-sourced DMSP is a powerful chemoattractant for a diverse range of marine organisms, including bacteria, phototrophic and heterotrophic protists, and fish [14, 35, 36]. However, it has also been suggested to act as a grazing deterrent for some herbivorous protists in experiments with bulk additions [29, 33] and when DMSP release from microalgae is triggered under light stress [37]. Thus, the DMSP chemotactic role in grazing is still under examination. DMS is well-known for triggering searching behaviour in birds, turtles, and seals [36, 38, 39], yet its chemoattraction for microbial plankton, particularly protists, appears to be weaker compared to DMSP [14, 40, 41]. In zooplankton, DMS has shown searching and grazing-enhancing effects [42, 43]. Regarding acrylate, while it was initially postulated as a predator repellent [44], actual evidence supporting this notion remains scarce [41].

In this study, we aimed to explore the chemotactic response of three herbivorous dinoflagellates —*Karlodinium armiger*, *Oxyrrhis marina*, and *Gyrodinium dominans*—to variable microscale gradients of DMSP, DMS, and acrylate. To achieve this, we used a modified microcapillary assay [40, 45], accompanied by video recording and state-of-the-art image analysis tools. The three microzooplankters were selected for their distinct trophic modes: *K. armiger* is a mixotroph that combines heterotrophy and photosynthesis [46], *O. marina* is an efficient phagotroph and osmotroph [47], and *G. dominans* is a strict phagotroph [48]. All three species are known consumers of DMSP producers [6, 37, 46, 49, 50] but vary in habitat preference and feeding behaviours. While *O. marina* preferentially occurs in intertidal pools, salt marshes, and embayments [51], *G. dominans* and *K. armiger* thrive in coastal and open ocean waters, with *G. dominans* even found in a dinoflagellate red tide [52] and in an *Emiliania huxleyi* bloom [53]. *Karlodinium armiger* diet includes a wide range of prey sizes, including large inert particles where DMSP may accumulate [28, 54].

## Materials and methods

### Experimental cultures and artificial seawater

All three dinoflagellate cultures (*O. marina* ICM-ZOO-OM001*, G. dominans* ICM-ZOO-GD001, and *K. armiger* ICM-ZOO-KA001) were originally isolated from the NW Mediterranean by A. Calbet in 1996, 2011, and 2013, respectively. *Rhodomonas salina* strain K-0294 was obtained from the NORCCA (The Norwegian Culture of Collection of Algae) and cultured in the Institute of Marine Sciences (ICM-CSIC) since 2018. We maintained the dinoflagellate cultures with autoclaved filtered seawater (FSW) on a 14:10 h light to dark cycle at 19°C, 50 μE m^−2^ s^−1^ of light, and with a *R. salina* diet. The prey culture was kept in FSW supplemented with f/2 medium [55] under the same conditions as the microzooplankton cultures, but with a higher light intensity (100 μE m^−2^ s^−1^). The cell concentration of both the grazer and prey populations were monitored regularly with a Multisizer 3 Coulter Counter (Beckman Coulter). The prey used to feed *O. marina* was previously concentrated by centrifugation at 1000 × g for 10 min. Prior to the experiments, the dinoflagellate cultures were starved until the majority were vacuole depleted and no prey were present in the culture. Dilutions of the cultures were made as needed to achieve the required microzooplankton concentration (8E+03 cells ml^−1^ for *K. armiger* and 2E+04 cells ml^−1^ for *O. marina* and *G. dominans)*. For the experiments we used artificial seawater (ASW) both as control and dilution medium for the substrates (Supplementary Information).

### Microcapillary assay

**Figure 1:**
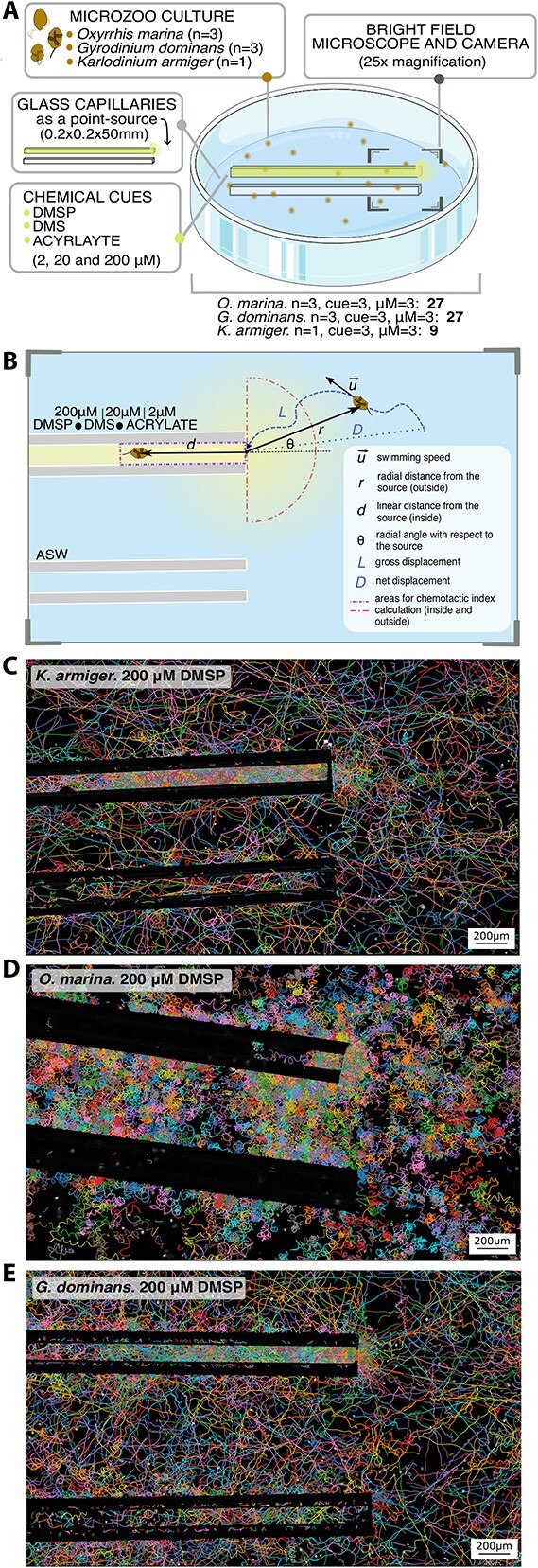
**The microcapillary assays**. (**a**) Diagram of the assay. (**b**) Schematic representation of the experimental setup and parametrization used in the trajectory analysis. The dashed lines represent the two areas of interest: inside the capillary, and the area of influence outside the capillary. (**c–e**) Cell tracks of *K. armiger* (c), *O. marina* (d), and *G. dominans* (e) recorded during the first 900 frames (30 s) of the interval of maximum chemoresponse in the 200 μM DMSP assays.

To assess the chemotactic potential of DMSP, DMS, and acrylate, we employed a microcapillary technique [56] previously modified [57] and adapted to *O. marina* cultures [36, 41]. The microcapillaries (CM Scientific) were square-shaped and manufactured in borosilicate glass, 0.2 mm wide per 50 mm long, and with a wall thickness of 0.100 mm. The three dinoflagellates were tested against three chemicals: DMSP (TCI America), DMS (Sigma-Aldrich), and sodium acrylate (Sigma-Aldrich), each in three concentrations: 2, 20, and 200 μM. Solutions of each substrate were prepared on the day of the experiment by serial dilutions with ASW. The experiment consisted of incubations of microcapillary pairs filled by capillary action with solutions of the substrate of interest, and ASW as the control solution. The capillaries were placed on the bottom of a sterile Petri dish (8.5 cm diameter) filled with 15 ml of the culture to be tested (Fig. 1a). The assays were recorded using a Leica DMil bright field microscope with 25× magnification and the Leica MC170HD camera acquiring images at 30 frames per second. The videos lasted 10 min except for the 200 μM DMSP assays with *G. dominans* and *O. marina* cultures, where the experiment was stopped after 5 min. Each chemical and concentration combination was tested in triplicates, except for *K. armiger* experiments, which were tested only once. Minor adjustments to the setup were made for *K. armiger* assays, where the capillaries were cut in half and placed in smaller (5 cm) Petri dishes. We handle the capillaries with tweezers to avoid contamination.

### Image analysis and trajectory tracking

Each video was pre-processed by subtracting the background from each frame. The background image was used to extract the coordinates of the capillaries’ internal perimeter and of the central point of the capillary entrance, set as the origin of the coordinates system (Fig. 1b). Individual cells were located in every frame and their trajectories were reconstructed using the Python package Trackpy 0.5 (10.5281/zenodo.4682814) (Fig. 1c–e).

### Cell accumulation and chemotactic index calculations

**Figure 2:**
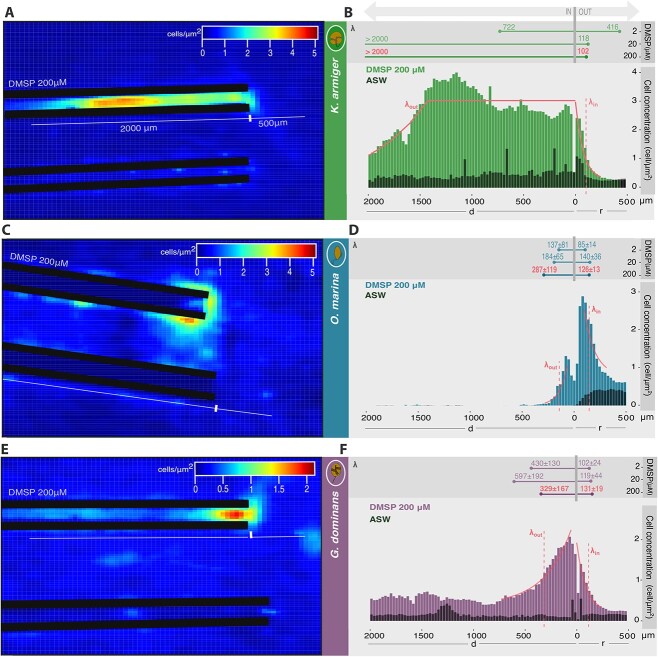
**Cell density distributions in the microcapillary assays.** Cell density distributions of *K. armiger* (**a, b**), *O. marina* (**c, d**), and *G. dominans* (**e, f**) in microcapillary assays with 200 mM DMSP, depicted as colormaps (**a, c, e**) and histograms (**b, d, f**). Note that the capillary with the cue is always compared with a control without the cue. The control capillary is always the one at the bottom in the colormaps and is represented in the histograms as black bars. The curves over the bars show the exponential fit on the accumulation profile, and the dashed lines the depth of exploration inside the capillary (λ_in_) and the size of the cell patch outside of it (λ_out_) for that replicate. The upper panels of the histograms illustrate all λ values, specified for each DMSP concentration. The exponential decay constant λ is interpreted as the typical scale of accumulation. Symbols: d = linear distance, inside; r = radial distance, outside.


The average concentration of cells was determined during the total recording period and across all recording area, highlighting the locations most frequently visited by each organism (Fig. 2a,c,e). The concentration profiles were calculated in two areas of space where cell accumulation was observed: inside the capillary and in the external semi-circular region centred in the origin (Figs 1b and 2b,d,f). Reflecting the symmetry of the infochemical gradient, the concentration profiles were calculated along the capillary axis inside and radially outside. The concentrations profiles were fitted to a decaying exponential function:

$$ C(d)=a\ast \exp \left(-\frac{d}{\lambda_{in}}\right)+b\ inside\ the\ capillary $$

(1) \begin{equation*} C(r)=a\ast \exp \left(-\frac{r}{\lambda_{out}}\right)+b\ outside\ the\ capillary \end{equation*}

The exponential decay constants ${\mathrm{\lambda}}_{in}$ and ${\mathrm{\lambda}}_{out}$ quantify, respectively, the depth of exploration inside the capillary and the extension of the patch at the capillary entrance. In the case of *K. armiger* with DMSP 20 and 200 μM, the concentration *C(d)* was fitted to a piecewise function composed of a constant plateau preceding the exponential decay (Fig. 2b, d, f). For the three organisms, the values of ${\mathrm{\lambda}}_{in}$and ${\mathrm{\lambda}}_{out}$ are averaged between experimental repetitions.

Maximum chemotactic indices *Ic_max_* were calculated both inside and at the entrance of the capillaries, as:


(2) \begin{equation*} {Ic}_{max}=\frac{{\overline{C}}_S}{{\overline{C}}_C} \end{equation*}

where *C_S_* is the average cell concentration in the substrate (cue)-filled capillary during the 2 min around the maximum cell accumulation, and *C_C_* is the average concentration inside the control capillary over the entire assay (Supplementary Information, Supplementary Figure S6). Inside the capillaries, the concentrations are calculated in the rectangular area within a distance ${\mathrm{\lambda}}_{in}$ from the entrance, outside the capillaries on the semi-circular region of radius ${\mathrm{\lambda}}_{out}$. Accordingly, two chemotactic indexes are finally reported: one inside and one outside the capillary (for further details see Supplementary Information). Significant differences in cell concentration between the cue and the control capillary were evaluated with the nonparametric Kruskal–Wallis test prior to computing the *Ic_max_* (Supplementary Tables S4–S6). When nonsignificant differences were found (*P* > 0.01), *Ic_max_* was considered equal to 1 (neutral, no response). All statistical analyses were conducted in R v4.0.2 (R Development Core Team, 2021).

### Swimming behaviour analyses

The swimming behaviour in response to a DMSP patch was characterized in terms of swimming speed and trajectory straightness. The trajectory straightness *S* was evaluated for trajectories ingoing and outgoing the infochemical source and for trajectories unaffected by the chemical (marked as “neutral zone”). *S* is calculated as [58]:


(3) \begin{equation*} S=D/L \end{equation*}

where *D* is the distance in the 2D plane between the initial point and the end point of a trajectory and *L* is the path length, defined as $\sum_{i=1}^N{l}_i$for a trajectory made of N steps of length ${l}_i$. Hence, $S=1$ for a totally straight trajectory, and $S\ll 1$ for a very convoluted path.

Incoming, outgoing, and unaffected trajectories were computed within circular coronas with a width of 140 μm, corresponding to roughly seven times the approximate body length of the cell (20 μm). For ingoing and outgoing trajectories, *S* was calculated on a portion of the trajectory fully contained in the diffusing infochemical patch (up to 200 μm after 60 s of recording). *S* for trajectories unaffected by the chemical was calculated during the first 60 s of recording at a distance >400 μm. Given the inherent dependence of *S* on the trajectory length, a robustness test was conducted to evaluate its stability across a diverse range of trajectory lengths (Supplementary Information, Supplementary Figure S7). The data used for *S* calculations were derived from assays with 20 and 200 μM DMSP.

### ^35^S-DMSP uptake experiments

Four aliquots of starved cultures of each organism were prepared in tissue culture flasks of 70 ml volume (Becton Dickinson). The aliquots were treated with a combination of three antibiotics: kanamycin (1000 μg ml^−1^), neomycin (250 μg ml^−1^), and penicillin (1000 μg ml^−1^) the day before the experiment to reduce bacterial abundance and activity. Each set of aliquots included two experimental replicates, one killed with 10% glutaraldehyde to serve as blank and one kept in a radioactivity-free lab to follow cell concentration and volume with the Multisizer 3 Coulter Counter (Beckman Coulter). The first three aliquots were spiked with 20,000 dpm ml^−1^ of ^35^S-DMSP (specific activity of 1186 dpm fmol^−1^; final concentration of 17 pmol l^−1^) and incubated at 19°C with continuous light (67 μE m^−2^ s^−1^) for a maximum of 48 h. Although dissolved DMSP was not measured in the culture, very low concentrations were expected since neither the prey nor the grazer are known to produce DMSP in high concentrations [16]. Following a previously described protocol [59], sample aliquots were filtered through 3–5 μm (nominal pore) nitrocellulose filters, washed twice with FSW, and immersed in Ultima Gold cocktail for measurement with a Beckman scintillation counter. The filtrate was re-filtered through 0.2 μm (nominal pore) nitrocellulose filters and analysed to detect uptake of the radioisotope by the free-living bacteria that may have survived the antibiotic treatment. Uptake in the bacterial fraction represented <2% of the total radioisotope added. Chemically synthesized and purified ^35^S-DMSP was provided by Stephen D. Archer (Bigelow, Laboratory for Ocean Sciences, USA).

## Results

### Use of a microcapillary assay to create microscale substrate diffusion gradients from a point source

We introduced an experimental capillary with cue and a control capillary without cue into a culture of the herbivore protist, and video-recorded cell distributions and swimming tracks (Fig. 1a). This assay, based on previous works [36, 41], was designed to replicate the formation of a microscale (hundreds of μm spread) gradient of a target compound resulted from diffusion from a point source, such as a leaky or bursting cell or aggregate [1,2,45] (Supplementary Information, Supplementary Fig. S5). We observed the chemotactic response of individual motile cells to this gradient (Fig. 1b). Unlike the classical capillary assay, where chemotactic cells are identified based on a dual yes/no criterion after entering the capillary, our approach allowed us to study the swimming behaviour and cell distribution both inside the capillary and on the outer side of the entrance. In this study, we investigated the chemotactic responses of the dinoflagellates *K. armiger***,***O. marina,* and *G. dominans* to the phytoplankton-derived cues DMSP, DMS, and acrylate.

### Chemoattracting role of DMSP

Cell tracks around the cue and control capillaries showed accumulation of *K. armiger*, *O. marina,* and *G. dominans* in the proximity of the DMSP source and inside the DMSP-filled capillary (Fig. 1c–e). Each organism exhibited distinct swimming patterns in response to the cue. *Karlodinium armiger* and *G. dominans* displayed more straight trajectories and inspected the inside of the DMSP capillary. In contrast, *O. marina* swam in more circular and convoluted tracks and accumulated mainly at the entrance of the capillary. This behaviour is quantitatively depicted by the cell density colormaps (Fig. 2a,c,e and Supplementary Fig. S1) and the spatial profiles of cell densities inside and outside the capillaries (Fig. 2b,d,f and Supplementary Fig. S2). Because the observed accumulation profiles decreased exponentially from the tip in both inwards and outwards of the capillary, the depth of exploration inside the capillary (λ_in_) and the size of the cell patch outside of it (λ_out_) of each organism was quantified by fitting an exponential function to the cell distribution data (Fig. 2b,d,f, M&M). The three species accumulated above the control at the outer side of the entrance of the DMSP capillary, in patches of similar size (λ_out_ ~ 84–140 μm). The exception was *K. armiger* at 2 μM DMSP, which showed little chemotactic response, indistinguishable from the control, in the preliminary (only one replicate was conducted) results obtained. Different behaviours were observed inside the capillary for each species: *K. armiger* swam deep into the capillary (λ_in_ ~ 700–2300 μm) with higher DMSP concentration; *G. dominans* accumulated both at the outer and inner sides of the capillary entrance, exploring part of the capillary (λ_in_ ~ 330–600 μm); and *O. marina* aggregated preferentially around the outer side of the entrance of the capillary and scarcely explored inside it (λ_in_ ~ 130–290 μm).

Cell accumulations over time inside the DMSP capillary also differed between organisms: *K. armiger* and *O. marina* showed steady long-term accumulation during the incubation, while *G. dominans* presented a fluctuating, up-and-down pattern in cell concentration (Supplementary Fig. S3). Consequently, regression-based long-term accumulation rates were positive for *O. marina*, positive but weaker for *K. armiger,* and slightly negative for *G. dominans* (Supplementary Tables S1–S3).

**Figure 3:**
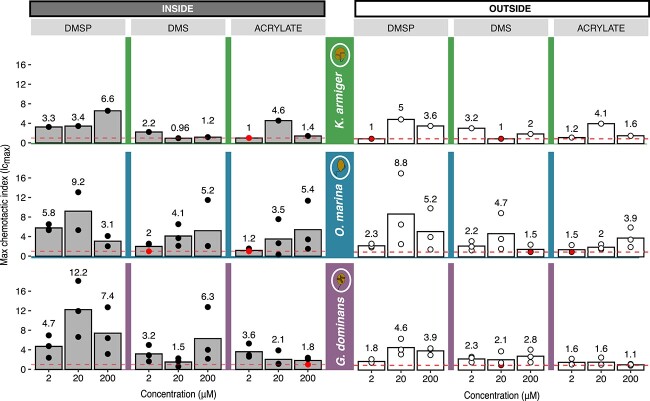
**Maximum chemotactic indices (*Ic***_***max***_ **) to DMSP, DMS, and acrylate.** The filled columns indicate the average *Ic_max_* (cell concentration in the substrate over cell concentration in the control) inside the capillary. The open columns indicate the average *Ic_max_* in the outside area. Average *Ic_max_* are given also numerically. Dots refer to the *Ic_max_* obtained in each replicate experiment. Nonsignificant differences (*P* > 0.01) in cell concentration that translated into *Ic_max_* = 1 are illustrated with filled dots on the dashed line. *Ic_max_* > 1 indicates positive chemoattraction, *Ic_max_* around 1 indicates neutral effect, and *Ic_max_* < 1 indicates chemotactic repulsion. The dashed line indicates *Ic_max_* = 1. Three concentrations (2, 20, and 200 μM) were tested per cue. The standard deviations of the averages are reported in Supplementary Tables S4–S6.

We further evaluated the chemoattractant potential of DMSP using the maximum chemotactic index (*Ic_max_*) (Fig. 3), which was considered different than 1 (no response) when mean cell counts inside or outside the cue capillary were significantly different from mean cell counts inside or outside the control capillary (Supplementary Tables S4–S6). Outside the capillary, the values of *Ic_max_* were higher than 1 for the three organisms at medium and high DMSP concentrations, with the highest indices (from 4.6 to 8.8) recorded at the intermediate concentration (20 μM). Inside the capillary, the values of *Ic_max_* for *G. dominans* were higher than outside at all DMSP concentrations, and the highest index of all experiments (12.2) was recorded for this organism at 20 μM. Both *O. marina* and *K. armiger* showed higher indexes inside than outside at 2 μM, and similar or slightly lower at 20 and 200 μM. *Oxyrrhis marina* and *G. dominans* showed the strongest inside chemoattraction with the intermediate DMSP concentration, whereas the strongest response of *K. armiger* was with 200 μM and there was no significant response with 2 μM. These results confirm that DMSP elicits positive chemotaxis (i.e. chemoattraction) in *O. marina* [14, 40], expand this to *G. dominans,* and suggest so for *K. armiger* too*.*

### Chemotactic responses to DMS

Cell density histograms of *K. armiger* showed positive response to 2 and 200 μM DMS, especially on the outer side of the capillary entrance (Supplementary Fig. S2). Like in the DMSP assays, *K. armiger* exhibited the widest cell dispersion inside the DMS capillary. However, notable positive response was only observed at the lowest DMS concentration (2 μM), both inside and outside the capillary (Fig. 3). Most *Ic_max_* values significantly higher than 1 were obtained with *O. marina* and *G. dominans* (Fig. 3). For *O. marina*, positive responses were observed in most experiments, yet with lower cell concentrations than with DMSP (Supplementary Fig. S3). *Ic_max_* values were highest at 20 μM outside and 20 and 200 μM inside the capillary (Fig. 3). *Gyrodinium dominans* showed *Ic_max_* values higher than 1 in most assays both inside and outside the capillary, albeit with lower cell concentrations compared to DMSP (Fig. 3 and Supplementary Fig. S3). Overall, DMS triggered a chemoattraction response, but weaker than that to DMSP under the experimental concentrations and conditions tested.

### Absence of chemotactic repulsion by acrylate

Like DMS, acrylate elicited scattered positive responses, which were weaker and less consistent than those observed for DMSP. We only observed attraction to acrylate above the control for *K. armiger* in the one test with 20 μM (Supplementary Fig. S3), which resulted in *Ic_max_* > 4 both inside and outside (Fig. 3). *Oxyrrhis marina* showed increased cell concentrations above the control in some replicates with 20 and 200 μM acrylate (inside) and 200 μM acrylate (outside) (Supplementary Fig. S3 and Fig. 3). *Gyrodinium dominans* only showed positive chemotaxis to acrylate inside the capillary with 2 μM (Fig. 3). While significant differences in cell counts between cue and control capillaries were observed for most incubations (Supplementary Tables S4–S6), mean *Ic_max_* remained close to 1 in most cases, particularly with increasing acrylate concentrations (Fig. 3). Notably, *Ic_max_* lower than 1 (indicative of negative chemotaxis) were not observed for any of the three dinoflagellates. In conclusion, acrylate elicited a generally neutral, sometimes attraction response, with no evidence of repulsion.

### Swimming behaviour of dinoflagellates in response to DMSP gradients

**Figure 4:**
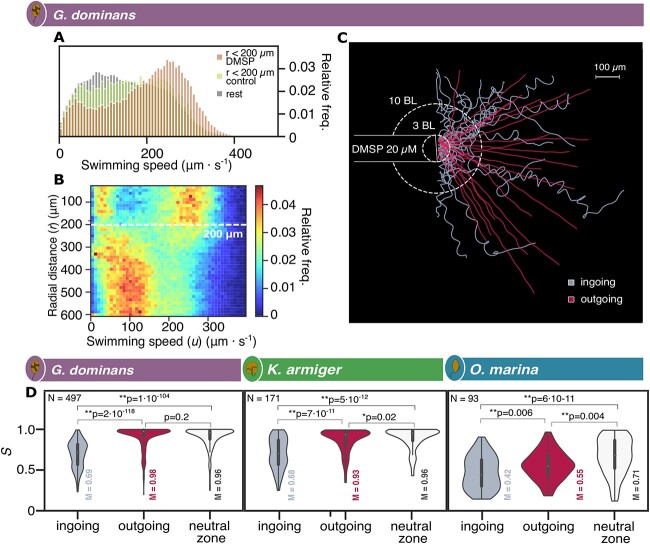
**Swimming behaviour response to DMSP.** (**A**) Relative frequencies of the instantaneous swimming speed (μm s^−1^) of *G. dominans* tested with DMSP 200 μM in three different zones: within a 200 μm radius from the DMSP source, within a 200 μm radius from the control capillary entrance, and in the remaining frame area. (**B**) Heat map of the relative frequencies of swimming speeds values versus radial distance (r: μm) in *G. dominans* cells trajectories tested with DMSP 200 μM. (**C**). Ingoing and outgoing *G. dominans* trajectories are distinguished, as tested with DMSP 20 μM. (**D**) Violin plots showing the data distribution of *S* values for ingoing (left), outgoing (middle), and background (right) trajectories. M represents the median value of each distribution and N is the number of trajectories analysed for each organism. Significant differences between *S*-values of the three zones also displayed in the figure with ** when *P* < 0.01 and * when *P* < 0.05. The section of the tracks employed in the analysis comprised between 3 and 10 BL (7BL = 140 μm) and are from 20 and 200 μM DMSP assays.

We observed changes in motility in the three organisms in presence of a DMSP gradient. Notably, a chemokinetic response (change in swimming speed) was specifically observed in *G. dominans*, where cells farther away from the DMSP source exhibited typical swimming speeds below 200 μm s^−1^, which increased to 300 μm s^−1^ near the DMSP source (Fig. 4a,b and Supplementary Fig. S4). This behaviour was consistently observed in all repetitions at intermediate (20 μM) and high (200 μM) concentrations of DMSP, except for one replicate at 20 μM (Supplementary Fig. S4). Conversely, no chemokinetic response was observed in either *K. armiger* or *O. marina.*

Regarding swimming trajectories, *G. dominans* and *K. armiger* showed a similar behavioural pattern: convoluted trajectories when approaching the DMSP source, and ballistic radial trajectories when exiting the capillary and moving away from it (Fig. 4c). This behavioural shift is illustrated by the straightness index *S* (Fig. 4d and Supplementary Table S7, Supplementary Fig. S8), which quantifies the net to gross displacement ratio of the trajectories (see Fig. 1b). The index *S* is thus a measure of the tortuosity of the path. Straight trajectories, where the organism takes the shortest path between two points, have values of *S* close to 1, while tortuous paths have low *S*. There was a noteworthy change in the median values of *S* between ingoing and outgoing trajectories for *G. dominans* (0.69 vs 0.98) and *K. armiger* (0.66 vs 0.98). The outgoing trajectories resembled background trajectories, i.e. those unaffected by the cue (Fig. 4d). This indicates that both organisms altered their swimming behaviour into a searching pattern (convoluted trajectories) when sensing an increasing gradient of DMSP, and reverted to their background ballistic swimming when leaving the gradient. For *O. marina*, the most notable difference was between the background swimming trajectories in the far (neutral) zone and those within the DMSP patch, regardless the in or out direction (Fig. 4d). Inside the patch the swimming became more convoluted, with median *S* of 0.42 (ingoing) and 0.55 (outgoing), compared to outside the patch (0.71).

### ^35^S-DMSP uptake by chemosensitive dinoflagellates

**Figure 5:**
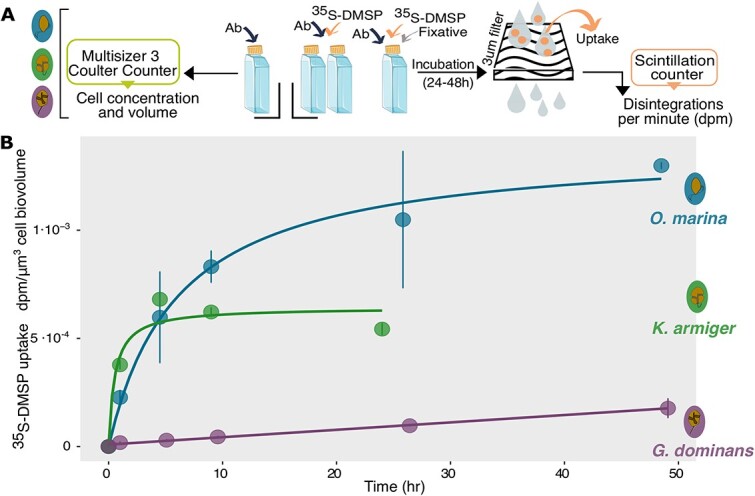
**Kinetics of DMSP uptake**. (**A**) Diagram of the uptake experiments (Ab = antibiotics). (**B**) Time course of ^35^S-DMSP uptake expressed as dpm incorporated per total cell biovolume (dpm μm^−3^). Standard deviation of replicate experiments (*n* = 2) is depicted by vertical lines.

To investigate whether the chemoattraction of our model organisms to DMSP was solely due to its role as an infochemical or also to its interest as a source of reduced sulphur, or both, we spiked the cultures with ^35^S-DMSP and monitored biological uptake over 24–48 h (Fig. 5). A previous study [59] showed the ability of *O. marina* to take up DMSP and assimilate its sulphur into biomass. For the sake of comparison between organisms, radioisotope uptake per cell (dpm cell^−1^) was normalized by cell volume (dpm μm^−3^), a proxy of biomass. The three dinoflagellates showed distinct uptake patterns. In the initial 5 h, *K. armiger* and *O. marina* exhibited uptake rates of 1.34E-04 dpm μm^−3^ h^−1^ and 1.26E-04 dpm μm^−3^ h^−1^, respectively. Although their uptake rates were comparable, their satiation levels differed significantly (*P* = 0.036): while *K. armiger*’s uptake rate levelled off at 6.81E-04 dpm μm^−3^, *O. marina*’s uptake reached 1.30E-03 dpm μm^−3^. Conversely, *G. dominans* uptake was much slower (5.36E-06 dpm μm^−3^ h^−1^) and had not yet levelled off after 48 h (Fig. 5). These results demonstrate that all three dinoflagellates are capable of DMSP uptake but differ largely in their uptake potential and efficiency.

## Discussion

In this study, we investigated the chemotactic responses of three marine herbivorous protists to microscale dissolved patches of three chemical cues. Our results confirmed a weak and variable chemoattractant role of DMS for *O. marina* [14, 40], and revealed this role for the first time in *G. dominans*. DMS, produced from algal DMSP due to oxidative stress, grazing, viral infection, and bacterial degradation [13, 18, 29], is expected to attract predators to physiologically compromised or aggregated prey. DMS production is typically accompanied by the production of acrylate. The role of acrylate as a grazing defence mechanism [41, 44] is questioned here based on the lack of observed repulsion responses. Since neither repeated attraction to low micromolar acrylate concentrations [44] nor repulsion by higher concentrations [40] were observed, acrylate cannot be postulated as an infochemical for the three dinoflagellates tested. However, each chemical has its own optimal range of response induction [40] and, although we tested a broad range of concentrations (2, 20, and 200 μM), it is still possible that we missed the range of maximal induction. Previous experiments with *O. marina* showed higher chemotactic indices with DMS concentrations lower than the ones tested here, but repulsion by acrylate was observed within the range we tested [40]. Conversely, both the visual inspection and the numerical data confirmed the strong and unquestionable chemoattractant role of DMSP. Chemoattraction to DMSP had been already reported for *O. marina* [6, 14, 40, 45] and now it is first described for *G. dominans* and *K. armiger*. DMSP induced strong aggregations within the patch, leading to significantly higher chemotactic indices (*Ic_max_*), with the highest values observed for *G. dominans.* The lack of experimental replication with *K. armiger* prevented adequate evaluation of the statistical significance of the results for this organism. In the context of the controversy about the role of DMSP as a grazing attractor [14] or deterrent [32, 60], our results support the attraction role and the idea that experimental bulk DMSP additions in grazing assays would mask natural microscale gradients and create a confounding landscape that hinders location and ingestion of the prey [14].

DMSP also prompted behavioural changes that reflected how each organism perceives, interprets, and uses DMSP. The observed alteration of the inwards swimming tracks in the three organisms illustrates the role of DMSP as a widespread foraging infochemical for marine protists [14]. In all cases, the elicited swimming response was consistent with helical klinotaxis, where helical paths are used to respond to and orientate in chemical gradients [61–63]. The more convoluted paths lead to shorter longitudinal displacements but improve the search by optimizing spatial sampling and orientation in gradients, especially when navigating an environment where the chemical signal is dim and the chemical plumes are stretched and advected by the flow [64]. Convoluted displacement patterns in the presence of prey (or prey signals) have been amply documented, from insects, fishes, and birds [36, 38, 65] to a variety of protists [14, 66], including marine dinoflagellates [6, 67–69]. This interpretation is reinforced by the observation of a return to ballistic swimming in the outgoing trajectories of *G. dominans* and *K. armiger* away from the source, suggesting that the behavioural (exploratory) alteration ceases once the DMSP-releasing target has been successfully located but there is no additional benefit in staying. Ecologically, we would expect *G. dominans* and *K. armiger* to quickly abandon a DMSP hotspot if they fail to find prey, as such hotspots might attract their potential predators. In the case of the osmotrophic *O. marina*, the DMSP-elicited alteration of the swimming behaviour persisted after the localization of the source.

*Oxyrrhis marina* exhibits high prey versatility with strong osmotrophic capabilities [47, 67]. Because its fast response to DMSP [14], we missed the immediate response in our experimental setup (<30 s), but subsequent behaviour confirmed its appetite for DMSP as a substrate. The low straightness index *S,* regardless of the displacement direction, indicated the interest of *O. marina* to remain in the patch. Its circular swimming behaviour, minimizing the chances of deviating away [62], aligns with its reported tendency of to remain in food patches for extended periods [6, 68]. Rapid aggregation near the DMSP source and steady long-term accumulation inside the DMSP capillary, yet with limited exploratory extent (low λ_in_), suggest that *O. marina* uses DMSP as an infochemical. When no prey are found, it remains in the patch to efficiently exploit DMSP as a substrate too, as supported by the high DMSP uptake rates with the highest satiation level observed in radioisotope assays. Thus, the short exploration depth inside the capillary reflects the high affinity of *O. marina* for DMSP uptake, which makes the dinoflagellate to change its swimming behaviour, reduce its exploratory activity, and remain within the substrate patch, likely exploiting its osmotrophic capability and avoiding extended energy expenditure.

*Gyrodinium dominans* is a typical phago-heterotroph that ingests prey by engulfment, lacking osmotrophic potential [48]. It showed a characteristic exploratory behaviour: tortuous trajectories and higher swimming speeds towards increasing DMSP concentrations. Analogous to what has been observed in other heterotrophic dinoflagellates after exposure to prey [69], the increase in velocity reflected *G. dominans* interpretation of DMSP as a prey signal, as the local 50% increase in swimming speed increased its probability to encounter prey [9]. In the absence of DMSP signal in the background conditions, lower swimming speeds reduced the risk of being encountered by predators [70]. The absence of prey and the inefficient use of DMSP as a substrate (very low uptake rates) resulted in no long-term accumulation in the capillary, leading to abandoning the DMSP patch and returning to regular ballistic swimming. This behaviour suggests that *G. dominans* and organisms alike use DMSP as a foraging infochemical; if the DMSP source cannot be engulfed (e.g. due to its cell size), *G. dominans* will shortly resume exploration elsewhere avoiding lingering in a potential hot spot for higher-trophic level predators.

*Karlodinium armiger* is a plastic mixotroph that can feed on a wide range of prey sizes, including particles much larger than itself, using peduncle feeding [46, 48]. Our ^35^S-DMSP uptake assays manifested its osmotrophic ability. DMSP sensing triggered a search behaviour with convoluted trajectories. The poorer detectability (higher *Ic_max_* in the ≥20 μM DMSP capillaries) and the search behaviour prompted a thorough inspection of the capillary that translated into the largest λ_in_ values. Inside the capillary, accumulation over time indicated fast uptake of DMSP; however, low satiation levels and the absence of prey elicited to leave the patch and return to ballistic trajectories. Overall, the data indicate that *K. armiger* is suited to detect high concentrations of DMSP and aggregate at the DMSP source, facilitating its proximity to DMSP-leaky particles for feeding via its peduncle. Confirmation of *K. armiger* behaviour would require further experimentation with multiple replicates.

Our findings have implications for our understanding and modelling of grazing in marine microbial plankton. While microzooplankton grazing rates are typically assessed and modelled based on cell sizes and encounter rates [9, 10], our work highlights the occurrence of selection based on chemotaxis, with preference for DMSP- or DMS-leaky prey. This has additional consequences for sulphur biogeochemical cycling. Grazing on DMSP-containing prey exerts a strong control on DMS concentration in the surface of the ocean and in the resulting emission of volatile sulphur to the atmosphere [30], with climate implications [13]. Yet, the ultimate effects of chemotaxis-driven grazing on plankton ecology and ocean biogeochemistry are far from straightforward. Despite showing that three dinoflagellates with distinct trophic modes are all attracted by algal cues, the diversity of the elicited behaviours complicates the picture. Chemotaxis to DMSP may enhance prey encounter and ingestion, but it also allows the protist to stay next to a feeding resource and change its local chemical environment. If the subsequent production of a secondary cue like DMS in turn attracts higher predators such as copepods or other mesozooplankton species that feed on the protist grazer [42], this may lead to tri-trophic interactions that will help relieve the grazing pressure on the DMSP producing prey [8, 71, 72].

Our study also demonstrates that chemotaxis favours protist aggregation on or around a DMSP source. In marine ecology, aggregation brings a trade-off of advantages and risks that is hard to resolve: contradictory results exist on the effects of aggregation on the probability of feeding and being predated [73, 74]. For heterotrophic dinoflagellates, enhanced feeding by aggregation on marine snow has been documented [75], and marine snow particles can be DMSP-rich [27]; therefore, chemotaxis-driven aggregation on marine snow may be a beneficial collective behaviour for micrograzers.

Natural point-sources cannot be expected to be composed of a single chemical cue. In the case of a chemical plume from a lysed or sloppy eaten cell, or a faecal pellet, it is expected that DMSP will concur with lower concentrations of DMS, and acrylate [42] as well as saccharides and other released metabolites [76]. Our experiments were designed to investigate the chemotactic properties of single compounds and the induced responses in particular strains. Future studies should address the potential synergies or counteractions of concurring cues [76], and more complex biological settings that better reproduce food web interactions [77]. Ultimately, understanding microbial food webs and their biogeochemical impacts requires a closer examination of the microscale, where key processes take place. The challenge remains of integrating and scaling up individual behaviours into community dynamics [78], food web functioning, and pelagic ecosystem services including element cycling and climate.

## Supplementary Material

170523_SI_wrae130

## Data Availability

The raw data and the secondary derived data have been placed in the Zenodo repository within the project “The distinctive chemotactic responses of three marine herbivore protists to DMSP and related compounds” in the following link: https://zenodo.org/communities/imedea_icm/records?q=&l=list&p=1&s=10&sort=newest The codes are accessible in Github in the following links and connected to the Zenodo databases: R scripts: https://github.com/medea95/Chemotaxis_experiments_R_scripts Python scripts: https://github.com/medea95/Chemotaxis_experiments_Python_scripts The cultures are in the ICM culture collection from which they can be ordered using the following link: https://www.icm.csic.es/ca/servei/cultius-de-plancton.
