# Supplementary material for: Distinctive chemotactic responses of three marine herbivore protists to DMSP and related compounds
Source: ISME J. 2024 Jul 12;18(1):wrae130. doi: 10.1093/ismejo/wrae130 (PMC11283757; doi:10.1093/ismejo/wrae130)
Supplement: 170523_SI_wrae130 [file 170523_si_wrae130.pdf]

SUPPLEMENTARY INFORMATION

The chemotactic behavioural response of marine herbivore protists to DMSP and related compounds

Queralt Güell-Bujons*[1,2], Medea Zanoli*[3], Idan Tuval[3], Albert Calbet[1], Rafel Simó[1¶]

[1]Institut de Ciències del Mar, ICM-CSIC, Barcelona, Catalonia, Spain
[2]Universitat Autònoma de Barcelona, Cerdanyola del Vallès, Catalonia, Spain.
[3]Institut Mediterrani d'Estudis Avançats, IMEDEA-CSIC, Esporles, Mallorca, Spain
* These authors contributed equally to this work

¶ Corresponding author: rsimo@icm.csic.es

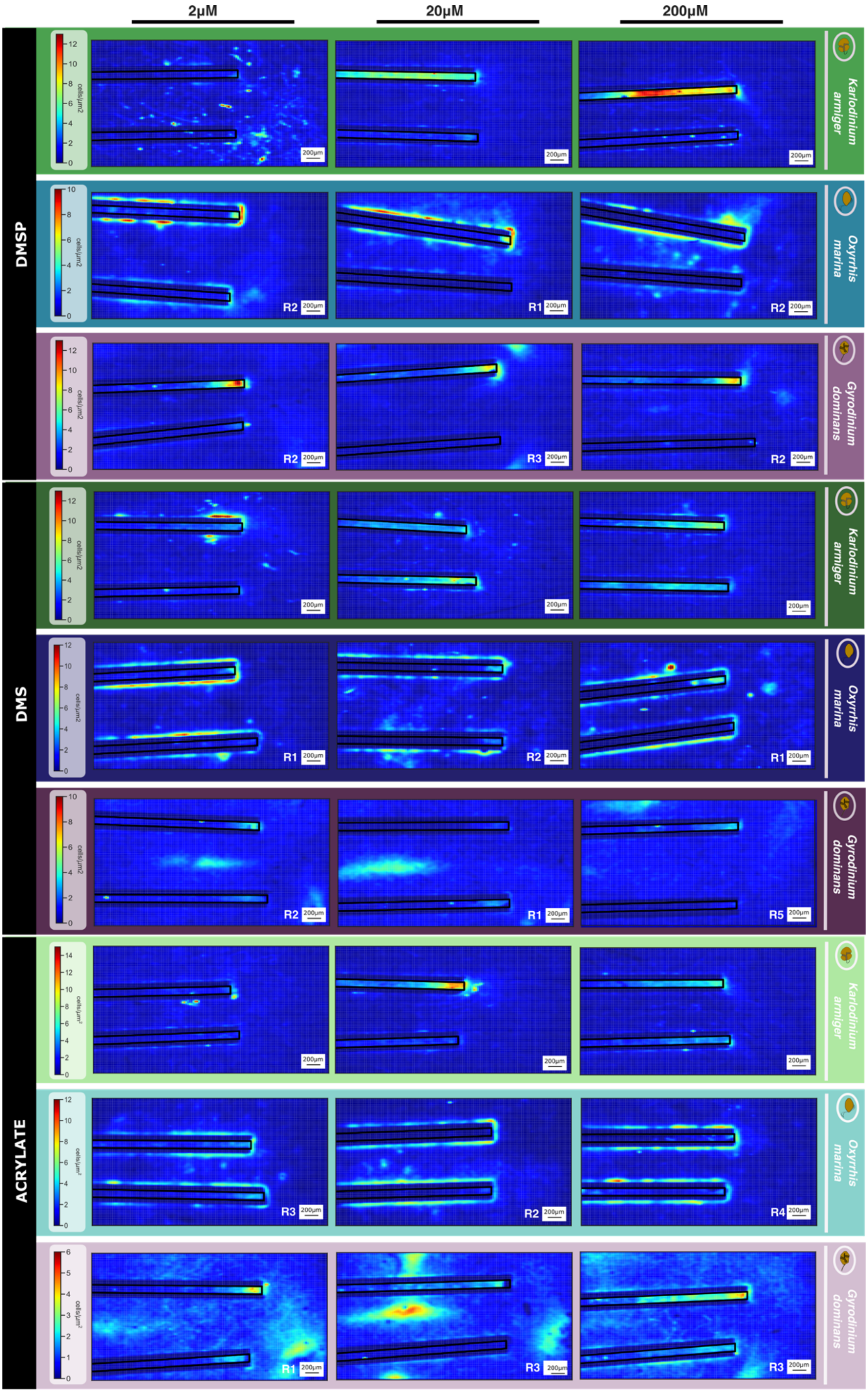

Supplementary Figure S1. **Cell density distributions in the microcapillary assays depicted by colormaps.** Cell density distributions of *K. armiger*, *O. marina*, and *G. dominans* in microcapillary assays. The figure shows the distributions in one replicate for each substrate and concentration. Cell density (cell μm^{-2}) values were normalized with background cell concentration.

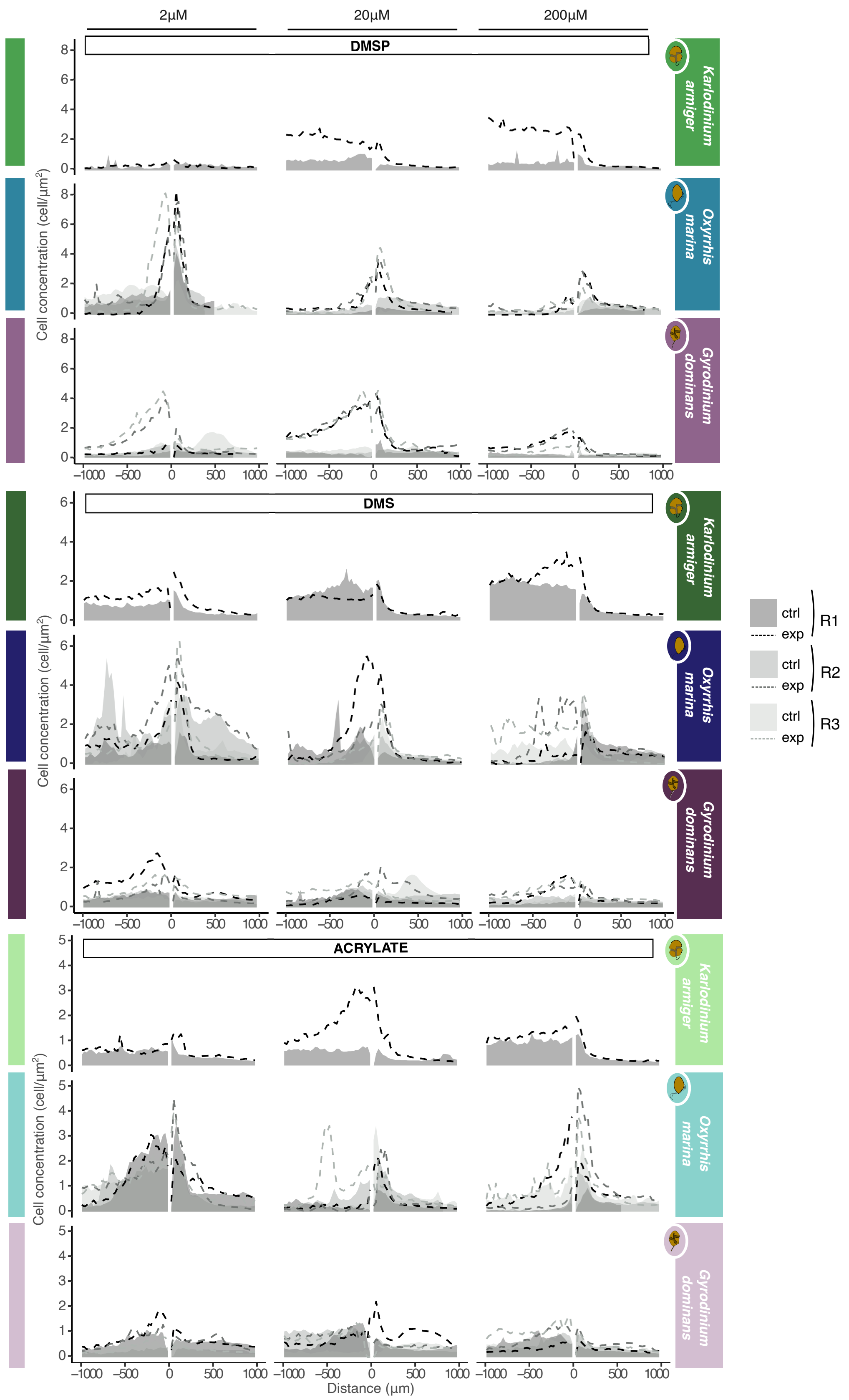

Supplementary Figure S2. Cell density distributions in the microcapillary assays depicted by histograms. Cell density distributions of *K. armiger, O. marina* and *G. dominans* in microcapillary assays depicted by histograms. The figure shows the distributions in all replicates, substrates, and concentrations. Note that the capillary with substrate (dashed lines) is always compared with a control without substrate (filled area). The 0 on the X axis of the histograms represents the capillary entrance, with the inside area on the left (negative distance) and the outside on the right (positive distance).

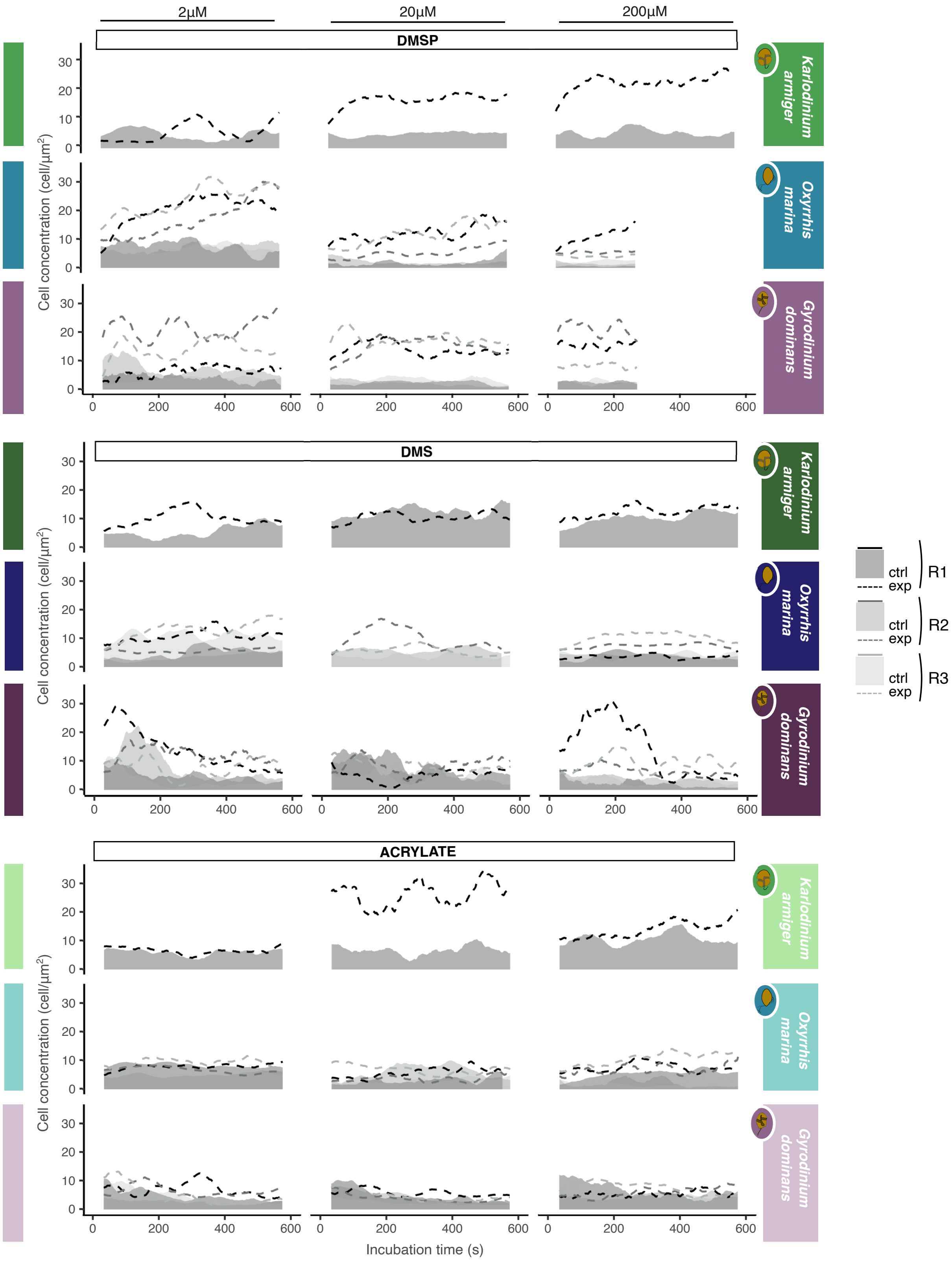

Supplementary Figure S3. Cell density temporal evolution. Total cell concentration in the two zones of interest was followed through all the incubation. Cell concentration inside and outside was totally calculated to observe temporal patterns in *K. armiger, O. marina*, and *G. dominans* microcapillary assays. The figure shows the total cell concentration in all replicates, substrates, and concentrations. Note that the capillary with substrate (dashed lines) is always compared with a control without substrate (filled area).

Supplementary Table S1. Linear regression results of *K. armiger* cell concentrations over time inside the capillaries. Accumulation in the DMSP capillary (cell µm^2 s^{-1}) was assessed by subtracting the slope in the control capillary (C) from the slope in the DMSP capillary (D). Std.err: standard error of the slope; p: probability of slope=0; Rep: replicate; err(D-C): propagated error of the slope subtraction.

K. armiger							
**Slope**	**Std.err**	**p**	**adj. R^2**	**Rep**	**Capillary**	**Slope(D–C)**	**err(D–C)**
**2 µM**							
**0.0085**	0.0006	1.36E-43	0.13	R1	DMSP	0.0127	0.0007
**-0.0042**	0.0003	4.80E-32	0.09	R1	CTRL		
**20 µM**							
**0.0019**	0.0002	1.46E-23	0.07	R1	DMSP	-0.0018	0.0002
**0.0037**	8.78E-05	8.21E-250	0.56	R1	CTRL		
**200 µM**							
**0.0051**	0.0003	5.43E-59	0.17	R1	DMSP	0.0051	0.0003
**-0.0007**	0.0003	0.0117	0.004	R1	CTRL		

Supplementary Table S2. Linear regression results of *O. marina* cell concentrations over time inside the capillaries. Accumulation in the DMSP capillary (cell µm^2 s^{-1}) was assessed by subtracting the slope in the control capillary (C) from the slope in the DMSP capillary (D). Std.err: standard error of the slope; p: probability of slope=0; Rep: replicate; err(D-C): propagated error of the slope subtraction; err: propagated error of the mean.

O. marina									
**Slope**	**Std.err.**	**p**	**adj. R^2**	**Rep**	**Capillary**	**Slope(D-C)**	**err(D-C)**	**mean**	**err.**
**2 µM**									
**0.0109**	0.0004	1.02E-120	0.32	R1	DMSP	0.0223	0.0005	0.0280	0.0003
**-0.0115**	0.0003	3.43E-205	0.48	R1	CTRL				
**0.0456**	0.0003	0.00E+00	0.93	R2	DMSP	0.0363	0.0004		
**0.0092**	0.0002	0.00E+00	0.70	R2	CTRL				
**0.0266**	0.0005	1.02E-310	0.64	R3	DMSP	0.0253	0.0006		
**0.0013**	0.0002	1.94E-09	0.02	R3	CTRL				
**20 µM**									
**0.0164**	0.0004	1.95E-267	0.58	R1	DMSP	0.0116	0.0004	0.0131	0.0002
**0.0048**	0.0002	4.46E-105	0.29	R1	CTRL				
**0.0101**	0.0002	0.00E+00	0.67	R2	DMSP	0.0112	0.0002		
**-0.0010**	0.0001	1.67E-39	0.12	R2	CTRL				
**0.0187**	0.0003	0.00E+00	0.67	R3	DMSP	0.0164	0.0003		
**0.0023**	0.0000	0.00E+00	0.76	R3	CTRL				
**200 µM**									
**0.0347**	0.0004	9.7E-322	0.95	R1	DMSP	0.0342	0.0004	0.0119	0.0002
**0.0005**	0.0001	6.33E-06	0.04	R1	CTRL				
**0.0018**	0.0003	6.51E-10	0.07	R2	DMSP	0.0028	0.0003		
**-0.0010**	0.0002	4.07E-08	0.06	R2	CTRL				
**0.0035**	0.0003	5.18E-38	0.28	R3	DMSP	-0.0013	0.0004		
**0.0048**	0.0003	1.18E-58	0.40	R3	CTRL				

Supplementary Table S3. Linear regression results of *G. dominans* cell concentrations over time inside the capillaries. Accumulation in the DMSP capillary (cell μm^2 s^{-1}) was assessed by subtracting the slope in the control capillary (C) from the slope in the DMSP capillary (D). Std.err: standard error of the slope; p: probability of slope=0; Rep: replicate; err(D-C): propagated error of the slope subtraction; err: propagated error of the mean.

G. dominans									
**Slope**	**Std.err.**	**p**	**adj. R^2**	**Rep**	**Capillary**	**Slope(D-C)**	**err(D-C)**	**mean**	**err.**
					**2 μM**				
**0.0045**	0.0003	4.00E-61	0.18	R1	DMSP	0.0117	0.0003	0.0101	0.0003
**-0.0072**	0.0001	2.02E-31	0.64	R1	CTRL				
**0.0115**	0.0006	8.62E-69	0.20	R2	DMSP	0.0168	0.0007		
**-0.0053**	0.0003	4.07E-56	0.16	R2	CTRL				
**0.0037**	0.0005	3.45E-12	0.03	R3	DMSP	0.0018	0.0005		
**0.0019**	0.0001	1.02E-59	0.17	R3	CTRL				
					**20 μM**				
**-0.0111**	0.0003	1.16E-19	0.46	R1	DMSP	-0.0094	0.0003	-0.0028	0.0002
**-0.0017**	0.0001	9.78E-35	0.10	R1	CTRL				
**-0.0050**	0.0004	4.30E-32	0.09	R2	DMSP	-0.0005	0.0004		
**-0.0045**	5E-05	0	0.83	R2	CTRL				
**0.0007**	0.0002	0.0032	0.005	R3	DMSP	0.0016	0.0003		
**-0.0009**	0.0001	4.34E-08	0.02	R3	CTRL				
					**200 μM**				
**0.0056**	0.0008	5.03E-13	0.10	R1	DMSP	0.0038	0.0008	-0.0054	0.0008
**0.0018**	0.0004	4.09E-07	0.08	R1	CTRL				
**-0.0110**	0.0019	1.98E-08	0.06	R2	DMSP	-0.0201	0.0019		
**0.0091**	0.0003	3.13E-14	0.72	R2	CTRL				
**-0.0076**	0.0007	4.35E-23	0.17	R3	DMSP	0.0001	0.0009		
**-0.0077**	0.0006	2.50E-36	0.27	R3	CTRL				

Instantaneous swimming speed. *G. dominans* showed a clear increase in swimming speed when tested with DMSP 20 μM and 200 μM (Supplementary Fig. S4). The histograms in the upper part of the panel display the observed relative frequency of swimming speeds in each repetition in 3 different zones: near the entrance of the DMSP filled capillary, near the control capillary and in the remaining frame area. Cells swimming close to the DMSP source ($r < 200$ μm) show a shift towards higher velocities (between 200 μm/s and 300 μm/s) in comparison to the rest of the cells. The colormaps in the lower part of each panel show the frequency of the observed swimming speed as a function of the distance from the DMSP source. A patch of cells swimming between 200 μm·s^{-1} and 300 μm·s^{-1} is distinguished in the upper part of the colormap corresponding to shorter radial distances. Contrastingly, cells further away from the source move typically below 150 μm·s^{-1}. The patch of fast swimming cells extends in most repetitions up to 200 μm from the DMSP source, except for R3 at 20 μM and R2 at 200 μM in which the patch extends up to approximately 400 μm. In most repetitions, we observe also a patch of slowly moving cells (< 70 μm·s^{-1}) at the entrance of the capillary. This behaviour is sometimes observed also in the control capillary (R2 at 2 μM, R4 at 20 μM, R2 at 20 μM) and may be caused by cells whose flagella became trapped at the pipette entrance. These cells slowly oscillate around the capillary entrance until they successfully break free and return to the bulk. A less clear chemokinetic behaviour is observed for repetitions at 2 μM. A mild chemokinetic effect is observed in R1, while in repetitions R2 and R3 the cells swim fast (between 200 μm·s^{-1} and 300 μm·s^{-1}) in the entire frame area and not only near the DMSP source as in the other repetitions.

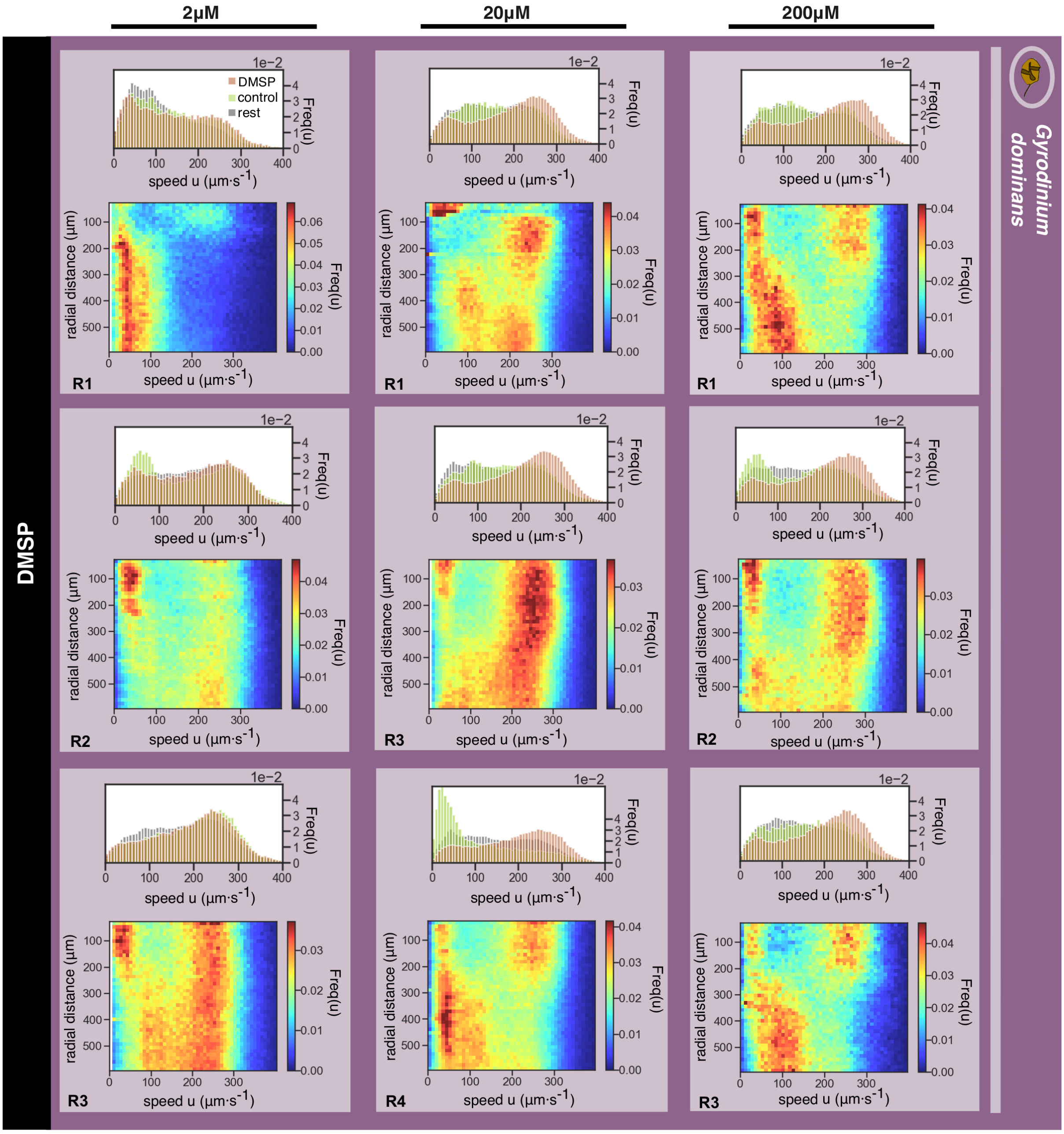

Supplementary Figure S4. Probability distribution of the instantaneous swimming speed of *G. dominans* at each DMSP concentration. Each panel contains two plots. The top chart compares the relative frequencies of the observed swimming speed within 3 different zones, represented in 3 colours: (i) orange: r < 200 μm from the entrance of the DMSP-filled capillary, (ii) green: r < 200 μm from the entrance of the control capillary and (iii) grey: the remaining frame area (r > 200 μm from both capillaries). The bottom chart is a colormap of the relative frequency of the instantaneous swimming speed at different radial distances from the entrance of the DMSP-filled capillary.

Supplementary Table S4. Median values, errors, and significant differences of cellular densities (cell/μm^2) from both capillaries (Cap. C=control and S=substrate-filled) in *K. armiger* incubations. Significant differences were evaluated with the nonparametric Kruskal-Wallis test. The statistical test was applied to each concentration (2-200µM), and cue (D=DMSP, M=DMS, A=ACRYLATE, Std.D=standard deviation, N=number of frames, Std.E= standard error).

K. armiger - INSIDE									
**Cap.**	**Mean (cell density)**	**Std.D**	**N**	**Std.E**	**p.value**	**Ic_{max}**	**Mean Ic_{max}**	**Std.E Ic_{max}**	**Sample**
C	2.37	1.22	1619	0.030	p<2.2E-16	3.26	3.26	0.00	D2_1
S	7.73	1.38	361	0.073					D2_1
C	3.95	0.54	1619	0.013	p<2.2E-16	3.44	3.44	0.00	D20_1
S	13.61	0.86	361	0.045					D20_1
C	2.91	0.81	1619	0.020	p<2.2E-16	6.57	6.57	0.00	D200_1
S	19.11	0.98	361	0.052					D200_1
C	3.33	1.42	1619	0.035	p<2.2E-16	2.23	2.23	0.00	M2_1
S	7.44	0.96	361	0.050					M2_1
C	8.42	1.01	1619	0.025	p=5.31E-06	0.96	0.96	0.00	M20_1
S	8.08	0.41	361	0.021					M20_1
C	7.56	1.39	1620	0.034	p<2.2E-16	1.18	1.18	0.00	M200_1
S	8.92	0.36	361	0.019					M200_1
C	3.86	1.06	1619	0.026	p=0.1568	1.00	1.00	0.00	A2_1
S	4.02	0.73	361	0.038					A2_1
C	4.67	1.15	1619	0.028	p<2.2E-16	4.55	4.55	0.00	A20_1
S	21.25	2.93	361	0.154					A20_1
C	6.49	1.29	1617	0.032	p<2.2E-16	1.42	1.42	0.00	A200_1
S	9.21	1.41	361	0.074					A200_1

K. armiger - OUTSIDE									
**Cap.**	**Mean (cell density)**	**Std.D**	**N**	**Std.E**	**p.value**	**Ic_{max}**	**Mean Ic_{max}**	**Std.E Ic_{max}**	**Sample**
C	2.48	0.83	1619	0.021	p=0.9997	1.00	1.00	0.00	D2_1
S	2.27	0.28	181	0.021					D2_1
C	1.00	0.33	1619	0.008	p<2.2E-16	4.96	4.96	0.00	D20_1
S	4.94	0.48	361	0.025					D20_1
C	2.43	0.94	1619	0.023	p<2.2E-16	3.62	3.62	0.00	D200_1
S	8.82	1.04	306	0.059					D200_1
C	2.28	0.87	1619	0.022	p<2.2E-16	3.15	3.15	0.00	M2_1
S	7.18	0.86	361	0.045					M2_1
C	4.04	1.19	1619	0.029	p=0.01899	1.00	1.00	0.00	M20_1
S	3.79	0.97	361	0.051					M20_1
C	2.82	0.69	1620	0.017	p<2.2E-16	1.98	1.98	0.00	M200_1
S	5.59	0.57	361	0.030					M200_1

C	2.10	0.36	1619	0.009	p<2.2E-16	1.24	1.24	0.00	A2_1
S	2.61	0.22	361	0.012					A2_1
C	2.09	0.74	1619	0.018	p<2.2E-16	4.09	4.09	0.00	A20_1
S	8.56	0.78	361	0.041					A20_1
C	4.23	1.55	1617	0.039	p<2.2E-16	1.62	1.62	0.00	A200_1
S	6.83	0.64	361	0.033					A200_1

Supplementary Table S5. Median values, errors, and significant differences of cellular densities (cell/μm^2) from both capillaries (Cap. C=control and S=substrate-filled) in *O. marina* incubations. Significant differences were evaluated with the nonparametric Kruskal-Wallis test. The statistical test was applied to each replicate (1-3), concentration (2-200µM), and cue (D=DMSP, M=DMS, A=ACRYLATE, Std.D=standard deviation, N=number of frames, Std.E= standard error).

O. marina **- INSIDE**									
**Cap.**	**Mean (cell density)**	**Std.D**	**N**	**Std.E**	**p.value**	**Ic_{max}**	**Mean Ic_{max}**	**Std.E Ic_{max}**	**Sample**
C	3.14	1.45	1620	0.036	p<2.2E-16	5.49	5.78	0.65	D2_1
S	17.26	1.46	361	0.077					D2_1
C	2.73	0.77	1619	0.019	p<2.2E-16	6.53			D2_2
S	17.83	2.25	361	0.119					D2_2
C	4.16	0.94	1620	0.023	p<2.2E-16	5.31			D2_3
S	22.12	1.61	361	0.085					D2_3
C	0.77	0.65	1619	0.016	p<2.2E-16	13.08	9.20	5.48	D20_1
S	10.07	1.74	361	0.091					D20_1
C	1.01	0.60	1619	0.015	p<2.2E-16	5.33			D20_2
S	5.38	0.84	361	0.044					D20_2
C	0.06	0.09	1619	0.002	p<2.2E-16	172.11			D20_3
S	9.50	0.73	361	0.038					D20_3
C	0.01	0.01	720	0.000	p<2.2E-16	1030.64	3.07	1.46	D200_1
S	7.04	1.42	361	0.075					D200_1
C	0.50	0.26	720	0.010	p<2.2E-16	4.11			D200_2
S	2.06	0.24	361	0.012					D200_2
C	0.96	0.23	720	0.008	p<2.2E-16	2.04			D200_3
S	1.96	0.34	361	0.018					D200_3
C	3.02	1.90	1619	0.047	p<2.2E-16	2.54	1.98	0.83	M2_1
S	7.68	1.17	361	0.062					M2_1
C	1.74	0.90	1620	0.022	p<2.2E-16	2.41			M2_2
S	4.18	0.42	361	0.022					M2_2
C	6.34	1.52	1618	0.038	p=0.01629	1.00			M2_3
S	6.58	1.37	361	0.072					M2_3
C	4.87	2.88	1620	0.072	p<2.2E-16	6.54	4.11	2.24	M20_1
S	31.85	2.10	361	0.110					M20_1
C	4.04	0.92	1541	0.024	p<2.2E-16	2.12			M20_2

S	8.54	0.82	361	0.043					M20_2
C	1.09	0.51	1619	0.013	p<2.2E-16	3.69			M20_3
S	4.03	0.27	361	0.014					M20_3
C	0.90	0.48	1620	0.012	p<2.2E-16	2.10	5.21	5.43	M200_1
S	1.88	0.34	361	0.018					M200_1
C	0.77	0.39	1620	0.010	p<2.2E-16	11.49			M200_2
S	8.88	0.12	361	0.006					M200_2
C	2.72	0.38	1620	0.010	p<2.2E-16	2.06			M200_3
S	5.58	0.12	361	0.006					M200_3
C	5.53	0.52	1620	0.013	p=0.05181	1.00	1.18	0.31	A2_1
S	5.61	0.73	361	0.039					A2_1
C	2.91	0.50	1619	0.013	p<2.2E-16	1.54			A2_2
S	4.49	0.33	361	0.017					A2_2
C	4.83	0.64	1618	0.016	p=0.02255	1.00			A2_3
S	4.94	0.35	361	0.018					A2_3
C	0.76	0.37	1549	0.009	p<2.2E-16	2.64	3.52	3.69	A20_1
S	2.01	0.61	361	0.032					A20_1
C	3.76	1.97	1620	0.049	p<2.2E-16	0.35			A20_2
S	1.33	0.18	361	0.009					A20_2
C	0.62	0.34	1619	0.008	p<2.2E-16	7.57			A20_3
S	4.69	0.30	361	0.016					A20_3
C	1.06	1.08	1620	0.027	p<2.2E-16	3.44	5.43	5.21	A200_1
S	3.63	1.22	361	0.064					A200_1
C	0.61	0.41	1620	0.010	p<2.2E-16	11.34			A200_2
S	6.94	0.98	361	0.051					A200_2
C	2.57	0.68	1620	0.017	p<2.2E-16	1.51			A200_3
S	3.88	0.67	361	0.035					A200_3

O. marina - OUTSIDE									
**Cap.**	**Mean (cell density)**	**Std.D**	**N**	**Std.E**	**p.value**	**Ic_{max}**	**Mean Ic_{max}**	**Std.E Ic_{max}**	**Sample**
C	4.39	0.77	1620	0.019	p<2.2E-16	1.95	2.27	0.38	D2_1
S	8.56	0.62	361	0.033					D2_1
C	4.32	1.00	1619	0.025	p<2.2E-16	2.18			D2_2
S	9.40	0.72	361	0.038					D2_2
C	2.80	0.84	1620	0.021	p<2.2E-16	2.69			D2_3
S	7.53	0.84	187	0.062					D2_3
C	0.99	0.59	1619	0.015	p<2.2E-16	6.63	8.77	7.48	D20_1
S	6.55	0.48	361	0.025					D20_1
C	1.07	0.30	1619	0.007	p<2.2E-16	2.60			D20_2
S	2.79	0.12	361	0.006					D20_2
C	0.37	0.31	1619	0.008	p<2.2E-16	17.09			D20_3

S	6.35	0.85	361	0.045					D20_3
C	0.60	0.14	720	0.005	p<2.2E-16	9.93	5.17	4.31	D200_1
S	5.96	0.54	361	0.028					D200_1
C	0.92	0.20	720	0.008	p<2.2E-16	4.06			D200_2
S	3.72	0.35	361	0.018					D200_2
C	1.38	0.26	720	0.010	p<2.2E-16	1.53			D200_3
S	2.11	0.17	361	0.009					D200_3
C	1.89	0.37	1619	0.009	p<2.2E-16	3.21	2.21	0.94	M2_1
S	6.07	0.43	361	0.023					M2_1
C	1.79	0.41	1620	0.010	p<2.2E-16	1.33			M2_2
S	2.39	0.06	361	0.003					M2_2
C	4.74	0.76	1618	0.019	p<2.2E-16	2.10			M2_3
S	9.94	0.37	361	0.020					M2_3
C	1.19	0.50	1620	0.012	p<2.2E-16	8.95	4.75	3.77	M20_1
S	10.67	0.35	361	0.018					M20_1
C	1.97	0.67	1541	0.017	p<2.2E-16	3.67			M20_2
S	7.23	0.91	361	0.048					M20_2
C	2.57	0.48	1619	0.012	p<2.2E-16	1.63			M20_3
S	4.20	0.13	213	0.009					M20_3
C	2.86	0.99	1620	0.025	p=8.83E-10	1.09	1.53	0.84	M200_1
S	3.10	0.40	187	0.029					M200_1
C	3.44	0.77	1620	0.019	p=0.05444	1.00			M200_2
S	3.55	0.30	361	0.016					M200_2
C	1.34	0.30	1620	0.007	p<2.2E-16	2.50			M200_3
S	3.35	0.28	361	0.015					M200_3
C	2.83	0.31	1620	0.008	p=0.842	1.00	1.47	0.82	A2_1
S	2.80	0.11	361	0.006					A2_1
C	2.17	0.35	1619	0.009	p<2.2E-16	1.00			A2_2
S	4.93	0.09	181	0.007					A2_2
C	2.20	0.51	1618	0.013	p<2.2E-16	2.41			A2_3
S	5.31	0.63	361	0.033					A2_3
C	2.25	0.82	1549	0.021	p<2.2E-16	2.57	1.97	0.53	A20_1
S	5.78	0.83	361	0.044					A20_1
C	2.50	0.84	1620	0.021	p<2.2E-16	1.57			A20_2
S	3.92	0.73	334	0.040					A20_2
C	2.18	0.19	1619	0.005	p<2.2E-16	1.77			A20_3
S	3.85	0.27	331	0.015					A20_3
C	0.99	0.31	1620	0.008	p<2.2E-16	6.02	3.85	2.03	A200_1
S	5.94	1.28	223	0.085					A200_1
C	1.65	0.61	1620	0.015	p<2.2E-16	3.53			A200_2
S	5.84	0.20	361	0.010					A200_2

C	2.81	0.95	1620	0.024	p<2.2E-16	2.00			A200_3
S	5.62	0.65	361	0.034					A200_3

Supplementary Table S6. Median values, errors, and significant differences of cellular densities (cell/μm²) from both capillaries (Cap. C=control and S=substrate-filled) in *G. dominans* incubations. Significant differences were evaluated with the nonparametric Kruskal-Wallis test. The statistical test was applied to each replicate (1-3), concentration (2-200μM), and cue (D=DMSP, M=DMS, A=ACRYLATE, Std.D=standard deviation, N=number of frames, Std.E= standard error).

***G. dominans* - INSIDE**									
**Cap.**	**Mean (cell density)**	**Std.D**	**N**	**Std.E**	**p.value**	**Ic_{max}**	**Mean Ic_{max}**	**Std.E Ic_{max}**	**Sample**
C	2.47	0.96	1619	0.024	p<2.2E-16	2.39	4.70	2.28	D2_1
S	5.92	0.69	361	0.036					D2_1
C	4.19	2.28	1621	0.057	p<2.2E-16	4.75			D2_2
S	19.92	1.54	361	0.081					D2_2
C	2.25	0.61	1622	0.015	p<2.2E-16	6.95			D2_3
S	15.61	1.84	361	0.097					D2_3
C	1.12	0.44	1620	0.011	p<2.2E-16	11.93	12.22	5.73	D20_1
S	13.33	1.38	361	0.073					D20_1
C	2.34	0.79	1620	0.020	p<2.2E-16	6.64			D20_2
S	15.52	2.56	361	0.135					D20_2
C	0.69	0.16	1622	0.004	p<2.2E-16	18.10			D20_3
S	12.56	1.34	361	0.070					D20_3
C	1.94	0.36	722	0.013	p<2.2E-16	6.40	7.42	4.85	D200_1
S	12.41	0.76	361	0.040					D200_1
C	1.25	0.57	720	0.021	p<2.2E-16	12.70			D200_2
S	15.85	2.18	361	0.115					D200_2
C	1.86	0.49	721	0.018	p<2.2E-16	3.16			D200_3
S	5.87	0.87	361	0.046					D200_3
C	2.48	0.93	1619	0.023	p<2.2E-16	4.98	3.17	1.69	M2_1
S	12.33	1.63	361	0.086					M2_1
C	4.19	2.94	1619	0.073	p<2.2E-16	1.64			M2_2
S	6.90	1.46	361	0.077					M2_2
C	1.61	1.39	1619	0.035	p<2.2E-16	2.89			M2_3
S	4.65	1.29	361	0.068					M2_3
C	4.30	1.73	1619	0.043	p<2.2E-16	0.62	1.50	0.82	M20_1
S	2.66	0.64	361	0.034					M20_1
C	3.28	1.49	1618	0.037	p<2.2E-16	1.65			M20_2
S	5.42	0.94	361	0.050					M20_2
C	2.51	0.80	1622	0.020	p<2.2E-16	2.24			M20_3
S	5.64	0.51	361	0.027					M20_3
C	1.04	0.62	1619	0.015	p<2.2E-16	12.75	6.31	5.65	M200_1
S	13.28	1.61	361	0.085					M200_1

C	2.07	0.41	1620	0.010	p<2.2E-16	2.18			M200_2
S	4.50	0.48	361	0.025					M200_2
C	1.57	0.59	1619	0.015	p<2.2E-16	4.02			M200_3
S	6.31	1.01	361	0.053					M200_3
C	3.20	1.47	1619	0.036	p<2.2E-16	2.60	3.61	1.46	A2_1
S	8.31	1.37	361	0.072					A2_1
C	1.21	0.51	1619	0.013	p<2.2E-16	5.28			A2_2
S	6.38	0.95	361	0.050					A2_2
C	2.90	2.06	1618	0.051	p<2.2E-16	2.95			A2_3
S	8.56	1.97	361	0.103					A2_3
C	3.61	2.03	1619	0.050	p<2.2E-16	1.18	2.06	1.58	A20_1
S	4.26	0.62	361	0.033					A20_1
C	3.07	1.35	1619	0.034	p=1.81E-13	1.11			A20_2
S	3.42	0.69	361	0.036					A20_2
C	0.82	0.21	1620	0.005	p<2.2E-16	3.88			A20_3
S	3.17	0.24	361	0.013					A20_3
C	5.02	2.35	1620	0.058	p=0.06103	1.00	1.82	0.73	A200_1
S	4.81	1.13	361	0.059					A200_1
C	2.89	0.78	1619	0.019	p<2.2E-16	2.10			A200_2
S	6.07	0.99	361	0.052					A200_2
C	3.26	0.79	1619	0.020	p<2.2E-16	2.37			A200_3
S	7.71	0.53	361	0.028					A200_3

G. dominans - OUTSIDE									
**Cap.**	**Mean (cell density)**	**Std.D**	**N**	**Std.E**	**p.value**	**Ic_{max}**	**Mean Ic_{max}**	**Std.E Ic_{max}**	**Sample**
C	1.53	0.60	1619	0.015	p<2.2E-16	1.51	1.76	0.40	D2_1
S	2.31	0.23	361	0.012					D2_1
C	3.26	1.27	1621	0.031	p<2.2E-16	1.55			D2_2
S	5.07	0.54	361	0.029					D2_2
C	1.46	0.29	1622	0.007	p<2.2E-16	2.22			D2_3
S	3.24	0.49	264	0.030					D2_3
C	1.06	0.49	1620	0.012	p<2.2E-16	6.41	4.60	1.64	D20_1
S	6.77	0.18	361	0.010					D20_1
C	1.20	0.21	1620	0.005	p<2.2E-16	3.22			D20_2
S	3.87	0.36	361	0.019					D20_2
C	1.69	0.72	1622	0.018	p<2.2E-16	4.19			D20_3
S	7.09	0.32	181	0.024					D20_3
C	0.74	0.14	722	0.005	p<2.2E-16	4.28	3.93	0.75	D200_1
S	3.18	0.28	361	0.015					D200_1
C	1.03	0.61	720	0.023	p<2.2E-16	4.45			D200_2
S	4.56	0.50	361	0.027					D200_2

C	1.02	0.20	720	0.008	p<2.2E-16	3.07			D200_3
S	3.12	0.15	204	0.010					D200_3
C	1.27	0.27	1619	0.007	p<2.2E-16	1.60	2.27	0.60	M2_1
S	2.03	0.12	361	0.006					M2_1
C	0.99	0.38	1619	0.009	p<2.2E-16	2.73			M2_2
S	2.70	0.32	361	0.017					M2_2
C	1.01	0.52	1619	0.013	p<2.2E-16	2.50			M2_3
S	2.53	0.23	361	0.012					M2_3
C	1.78	0.68	1619	0.017	p=0.8633	1.00	2.12	1.51	M20_1
S	1.67	0.36	361	0.019					M20_1
C	1.17	0.53	1618	0.013	p<2.2E-16	3.84			M20_2
S	4.49	0.49	361	0.026					M20_2
C	1.40	0.41	1622	0.010	p<2.2E-16	1.53			M20_3
S	2.14	0.27	361	0.014					M20_3
C	0.81	0.42	1619	0.010	p<2.2E-16	4.18	2.84	1.29	M200_1
S	3.40	0.47	361	0.025					M200_1
C	1.12	0.24	1620	0.006	p<2.2E-16	2.73			M200_2
S	3.07	0.41	361	0.022					M200_2
C	1.27	0.19	1619	0.005	p<2.2E-16	1.60			M200_3
S	2.04	0.11	361	0.006					M200_3
C	1.57	0.52	1619	0.013	p<2.2E-16	1.28	1.58	0.64	A2_1
S	2.01	0.32	249	0.021					A2_1
C	1.37	0.35	1619	0.009	p<2.2E-16	2.32			A2_2
S	3.17	0.32	361	0.017					A2_2
C	1.76	0.51	1618	0.013	p=2.72E-14	1.15			A2_3
S	2.02	0.26	361	0.014					A2_3
C	1.32	0.31	1619	0.008	p<2.2E-16	2.57	1.61	0.83	A20_1
S	3.39	0.18	361	0.009					A20_1
C	1.24	0.39	1619	0.010	p<2.2E-16	1.16			A20_2
S	1.44	0.35	361	0.018					A20_2
C	1.49	0.40	1620	0.010	p<2.2E-16	1.11			A20_3
S	1.66	0.15	220	0.010					A20_3
C	1.57	0.42	1620	0.011	p<2.2E-16	1.25	1.10	0.16	A200_1
S	1.96	0.19	361	0.010					A200_1
C	1.78	0.26	1619	0.006	p=1.87E-09	0.93			A200_2
S	1.65	0.06	361	0.003					A200_2
C	1.83	0.20	1619	0.005	p<2.2E-16	1.14			A200_3
S	2.08	0.17	361	0.009					A200_3

EXTENDED MATERIALS & METHODS

Diffusion experiment from a capillary of squared cross-section. The experimental design required both capillaries in the same frame and thus they were placed in close proximity. Consequently, after some time the control capillary would be reached by the diffusing DMSCs plume from the test capillary. The typical timescale needed for the plume to reach the control capillary can be approximated as:

$$\tau \sim \frac{x^2}{4D}$$

where *D* is the diffusion coefficient of the solute and *x* the distance between the two capillaries. In the experiment, the distance between test and control capillaries was typically $x{\sim}1\ mm$. Knowing the diffusion coefficients of DMSP ($D_{DMSP}{\sim}6 * 10^{-6}\ cm^2/s$), DMS ($D_{DMS}{\sim}1.1 * 10^{-5}\ cm^2/s$) and acrylate ($D_{acrylate}{\sim}1.1 * 10^{-5}\ cm^2/s$), the times after which the plumes would reach the entrance of the control capillary were $\tau_{DMSP}{\sim}\ 8\ min$, $\tau_{DMS} = \tau_{acrylate} \sim 4\ min$, i.e., within the duration of most video recordings. Nevertheless, we expected that the presence of a continuous DMSCs source would maintain a much stronger gradient at the entrance of the test capillary for the whole duration of the experiment. To confirm the expected gradients, we recorded the diffusion process from a square-shaped *CM Scientific* microcapillary immersed in L1 medium for 20 minutes using Allura Red as solute (Supplementary Fig. S5). The strength of the Allura Red gradients during the recording time at the source and 1 mm away (points A and B in Supplementary Fig.1a) was quantified by image analysis. The diffusion constant of Allura Red ($D_{Allura\ Red}{\sim}5 * 10^{-6}\ cm^2/s$) is almost the same as that of DMSP and half those of DMS and acrylate. Accordingly, the Allura Red gradients measured 20 minutes after the start of the diffusion process will thus be comparable with those of DMSP after the same time and those of DMS and acrylate after 10 minutes. Gradients were calculated for a scalar field *S* ranging between 0 and 1 proportional to a color linear scale (0 = black pixel, no Allura Red, 1= gray pixel, max Allura Red concentration inside the capillary) in units of μm^{-1}. Gradients are calculated radially over a semicircle centered at the entrance of the capillary $\theta \in [-\pi/2, \pi/2]$ (point A), where $\theta = 0$ corresponds to the direction parallel to the capillary. The same was done at point B one millimeter away from A. The temporal evolution of the gradients is shown in the left panel of Supplementary Fig. 1b. The histograms (right panel Supplementary Fig. 1b) represent the discrete probability distribution of the gradients *dS/dr* integrated for all values of $\theta \in [-\pi/2, \pi/2]$ over the total recording time (sampled once every minute). The results confirm that strong gradients were maintained near the DMSCs source compared to the control capillary for all compounds (DMSP, DMS, acrylate) for the whole duration of a 10-min. experiment.

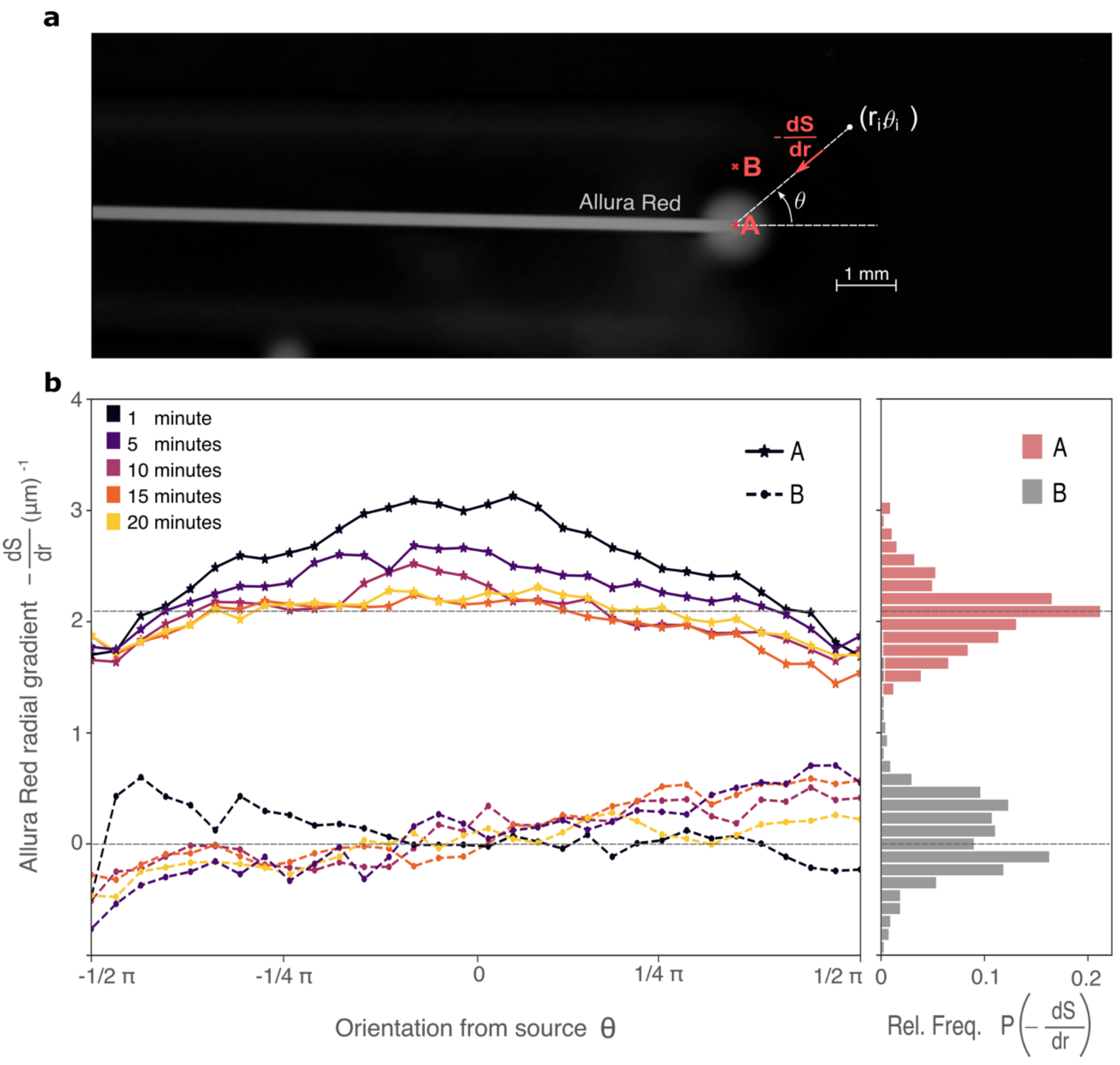

Supplementary Figure S5. Allura Red gradients as a proxy for DMSCs gradients (a) Diffusive plume from a square-shaped *CM Scientific* microcapillary filled with Allura Red in L1 medium at room temperature. The snapshot was taken 1 minute after the insertion of the Allura Red-filled capillary in the medium. The diffusion process was recorded over 20 minutes with an IDS camera. **(b)** Gradients of a scalar field *S* quantifying the Allura Red concentration. *S* is defined as a scalar number between 0 (black pixel, no Allura Red) and 1 (gray pixel, max Allura Red concentration inside the capillary). The gradients *dS/dr* are calculated radially in A and B over a semicircle $\theta \in [-\pi/2, \pi/2]$, where $\theta = 0$ corresponds to the direction parallel to the capillary. The left panel shows the radial distribution of the Allura-Red gradients after 1,5,10, 15 and 20 minutes from the addiction of the capillary to the medium. The right panel reports the probability distribution of the gradients measured in A and B, sampled every minute between 1 and 20 minutes starting from the addition of the capillary to the medium.

Artificial seawater (ASW). The ASW was elaborated by mixing and heating 1 l of distilled water with 43 g of sea salts (Sigma-Aldrich) and 1g of standard NaCl. The solution was filtered through GF/F to get rid of precipitated salts. The final salinity was 37.3 psu and the pH was adjusted to 7.9 by adding HCl. A second sterile filtration was conducted.

Chemotactic index calculation. To determine the maximum chemo-response observed during the experimental time, the *chemotactic index* I_c was calculated when cell accumulation in the substrate-filled capillary was at its peak (Fig. S6). To do so, the concentrations *C(x,t)* (inside the capillary) and *C(r,t)* (outside the capillary) were evaluated as a function of time and smoothed out with a 1-minute rolling average.

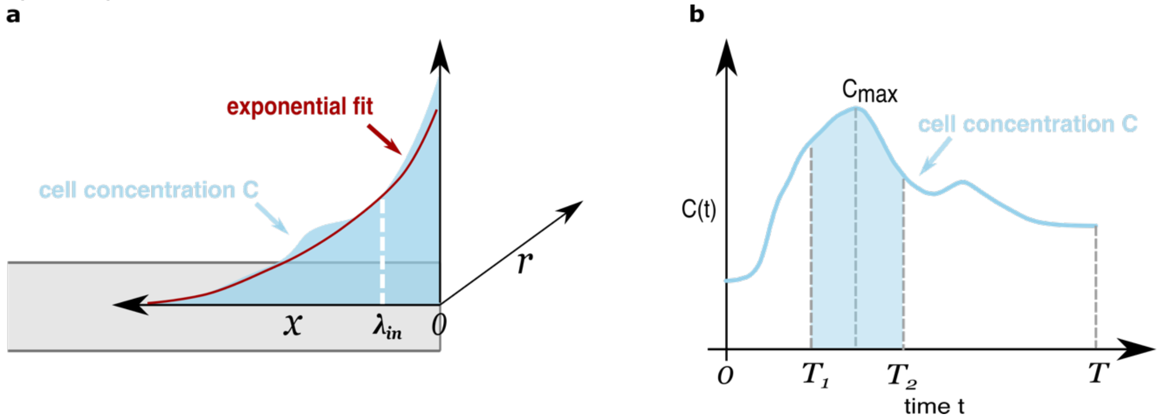

Supplementary Figure S6. Schematics for chemotactic index calculation. Variables used to calculate the chemotactic index inside the capillary. **(a)** The cell concentration profile *C(x)* is fitted to a decaying exponential with constant λ_{in}. The area of the capillary comprised between 0 and λ_{in} is the one taken into account to calculate the chemotactic index. **(b)** Temporal evolution of cell concentration *C(t)* in the area between 0 and λ_{in}. The experiment lasted a total time *T* (5 or 10 minutes depending on the repetition), and the times T_1 and T_2 embrace the 2-minute window of maximum chemoattraction, highlighted in blue.

The average cell concentration inside the substrate-filled capillary is then calculated as:

$$\overline{C}_S = \frac{1}{\lambda_{in}} \cdot \frac{1}{T_2 - T_1} \cdot \int_{T1}^{T2} dt \cdot \int_0^{\lambda_{in}} dx \; C(x,t)$$

where T_1 and T_2 are the extremes of the two-minute time window centered around the moment of maximum cell concentration, and λ_{in} is the exponential decay constant obtained from the fit of the concentration profile.

For the control, the average concentration is calculated as:

$$\overline{C}_C = \frac{1}{\lambda_{in}} \cdot \frac{1}{T} \cdot \int_0^{T} dt \cdot \int_0^{\lambda_{in}} dx \; C(x,t)$$

Where T is the total recording time. The average here is performed over the entire duration of the experiment to get a stable result for all repetitions and avoid null values of cell concentration.

Overall, I_c is calculated as:

$$I_C = \frac{\overline{C}_S}{\overline{C}_C}$$

The same calculation is carried out radially outside the capillaries, on a semi-circular region of radius λ_{out}. Accordingly, we get two chemotactic indexes: one inside and one outside the capillary.

Definition and robustness of the straightness index *S*. The straightness of a path is calculated as *S* = D/L, where D is the net 2D displacement and L the path length, has the drawback of being length-dependent. Considering a convoluted path and selecting a section of it, the calculated value of *S* for that particular section can vary depending on the length of the chosen section. For example, *S* would tend to 1 for a very short path, independently on how convoluted is the total trajectory.

To overcome the potential effect of the length of the track on S, for all trajectories we only used a fixed section of the path comprised between 3 and 10 body lengths (BL) calculated radially from the DMSP source after the first 1 minute of recording. Considering an average BL of 20 μm and a diffusion coefficient for DMSP of 0.6 E-5 cm2 s-1, the distance travelled by the diffusing solute in 1 minute is x ~ √(2Dt)= 270 μm. The value of S calculated within 10 BL (200 μm) thus corresponds to a trajectory fully contained within the DMSP patch. Trajectories that did not fully cross the 3 BL and 10 BL lines were discarded from the statistics. Non-continuous trajectories were allowed as long as the track cuts were shorter than 10% of the path length. To test the robustness of the index, *S* was calculated on trajectories covering a progressively greater distance (Supplementary Fig. S7). *S* was calculated on cells swimming towards or away from the DMSP source, covering a radial distance of 7, 9, 11, 13 and 15 body lengths (BL) with respect to the position of the entrance of the capillary. A cell is considered to be moving away from the DMSP source when its trajectory starts from less than 3 BL (60 μm) with respect to the capillary entrance and moves at least *N* BL far away from it (*N* = 7, 9, 11, 13, 15). The opposite is assumed for cells approaching the capillary. The straightness index was calculated also on the same range in an area unaffected by the chemoattractant (radial distance between 20 BL and 27 BL from the DMSP source during the first minute of recording), indicated as "neutral zone" (Supplementary Fig. S7). This area is located at 20 BL (400 μm) from the capillary. *S* in the neutral zone is calculated only on trajectories happening in the first minute of recording, to ensure that the area is unaffected by the diffusing DMSP plume. The nonparametric Kruskal-Wallis test, complemented with a post hoc Dunn's test, was employed to detect significant differences in S values amongst the three zones for each organism (Supplementary Table S7).

For all three dinoflagellates, S varied over a few hundredths in all areas, with no clear increasing or decreasing trend, suggesting that *S* is a robust parameter within the range studied (Supplementary Fig. S8). For *K. armiger*, the median *S* ranged between 0.65-0.68 for ingoing trajectories, 0.91-0.93 for outgoing trajectories, and 0.93-0.95 in the neutral zone. For *G. dominans*, the median *S* of incoming cells ranged between 0.66-0.70. Constant and close values were obtained for the outgoing trajectories (0.98) and in the neutral zone (0.96). For *O. marina*, the median *S* ranged between 0.45-0.50 for incoming cells, 0.53-0.55 for outgoing cells, and 0.71-0.76 in the neutral zone.

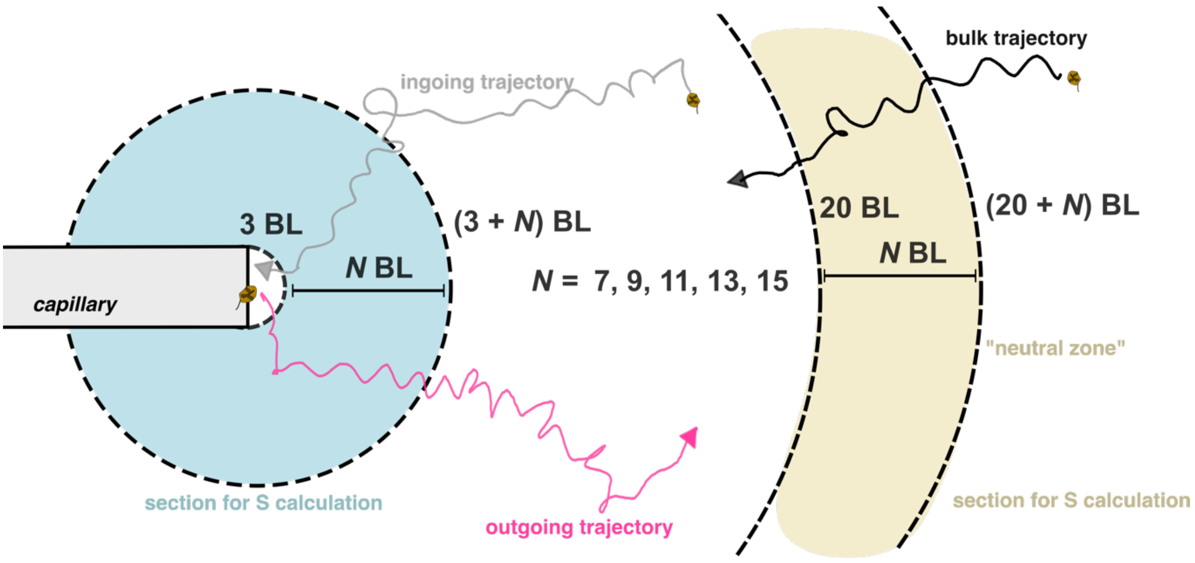

Supplementary Figure S7. Scheme of areas used for calculating the straightness index *S*. Trajectory straightness is calculated for 3 types of trajectories: trajectories going into the capillary from the bulk (gray), trajectories going back to the bulk after visiting the capillary (pink), and trajectories fully contained in the bulk (black). BL stands for "1 body length", corresponding to 20 µm. *S* is calculated on the trajectory section contained in two areas: the area near the capillary entrance (blue) and the area unaffected by the chemical (brown). The two areas are both circular coronas centered at the entrance of the capillary and whose smaller radius is set to, respectively, 3BL and 20BL from the capillary entrance. *S* is calculated on progressively larger areas by incrementing the size of the outer radius of each circular corona by *N* BL (*N* = 7, 9, 11, 13, 15).

Supplementary Table S7. Median *S* values and statistical results of the Kruskal-Wallis test complemented with the post-hoc Dunn's test for each organism. The statistical tests were applied to detect significant differences in *S* values between the 3 groups of trajectories (ingoing, outgoing and neutral zone). Each test and median is analyzed at different traveled distances calculated with a defined number (*N*) of body lengths (BL) to demonstrate the index (*S*) robustness.

	**Kruskal-Wallis test**		**Post-hoc Dunn's test (p-values)**			**Medina *S* values**		
	**H-statistic**	**p-value**	**Ingoing vs outgoing**	**Ingoing vs neutral**	**Outgoing vs neutral**	**Ingoing**	**Outgoing**	**Neutral zone**
K. armiger								
7 BL (140 µm)	136	3E-30	7E-11	5E-12	0.02	0.68	0.93	0.96
9 BL (180 µm)	113	2E-25	4E-18	6E-28	0.02	0.65	0.92	0.94
11 (220 µm)	102	4E-23	1E-18	6E-25	0.2	0.66	0.91	0.94
13 (260 µm)	91	1E-20	5E-16	5E-23	0.09	0.66	0.91	0.93
15 (300 µm)	92	1E-20	6E-17	3E-24	0.06	0.64	0.91	0.93
O.marina								
7 BL (140 µm)	41	1E-9	6E-3	6E-11	4E-3	0.42	0.55	0.71
9 BL (180 µm)	7	0.03	1	0.03	0.2	0.55	0.54	0.66

11 (220 μm)	4	0.1	0.6	0.1	1	0.55	0.62	0.66
13 (260 μm)	19	7E-05	0.008	2E-5	0.3	0.4	0.58	0.75
15 (300 μm)	10	6E-03	0.5	0.004	0.2	0.42	0.6	0.75
G.dominans								
7 BL (140 μm)	540	4E-118	2E-118	1E-104	0.2	0.69	0.98	0.96
9 BL (180 μm)	412	2E-90	2E-101	3E-82	0.01	0.67	0.98	0.95
11 (220 μm)	382	1E-83	4E-98	1E-80	0.01	0.66	0.98	0.96
13 (260 μm)	300	5E-66	4E-75	3E-63	0.1	0.64	0.98	0.96
15 (300 μm)	195	3E-43	2E-46	6E-39	0.3	0.68	0.98	0.96

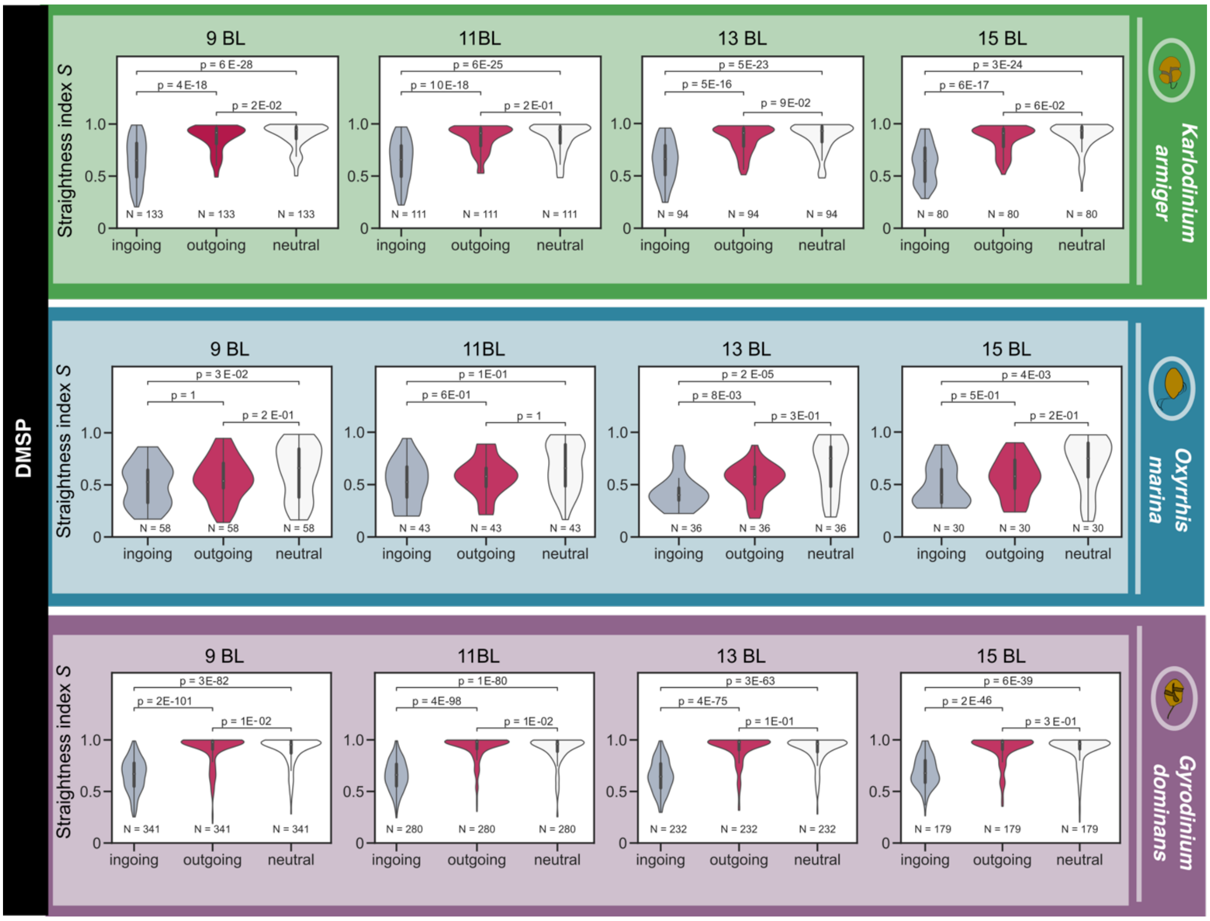

Supplementary Figure S8. Robustness test of the straightness index *S* for each organism. Each panel contains the violin plots with the *S* data distribution for trajectories covering a progressively greater distance (9, 11, 13, and 15 BL). Trajectories are divided into 3 types: trajectories approaching the DMSP source (ingoing – grey), trajectories moving away from the DMSP source (outgoing - magenta), and trajectories comprised in a neutral zone unaffected by DMSP (neutral – white). N refers to the number of trajectories analyzed in each set. Significant differences between the three zones can be detected by the p-values presented in each individual plot.
